# Identification of the Association Between Toll-Like Receptors and T-Cell Activation in Takayasu’s Arteritis

**DOI:** 10.3389/fimmu.2021.792901

**Published:** 2022-01-20

**Authors:** Yixiao Tian, Biqing Huang, Jing Li, Xinping Tian, Xiaofeng Zeng

**Affiliations:** ^1^ Department of Rheumatology and Clinical Immunology, Chinese Academy of Medical Sciences & Peking Union Medical College, Peking Union Medical College Hospital (PUMCH), Beijing, China; ^2^ National Clinical Research Center for Dermatologic and Immunologic Diseases (NCRC-DID), Ministry of Science & Technology, Beijing, China; ^3^ State Key Laboratory of Complex Severe and Rare Diseases, Peking Union Medical College Hospital (PUMCH), Beijing, China; ^4^ Key Laboratory of Rheumatology and Clinical Immunology, Ministry of Education, Beijing, China

**Keywords:** T-cell activation, Toll-like receptor, Takayasu’s arteritis, disease activity, co-stimulatory molecule, immune checkpoint, biomarker, miRNA - microRNA

## Abstract

To explore the relationships between Toll-like receptors (TLRs) and the activation and differentiation of T-cells in Takayasu’s arteritis (TAK), using real-time fluorescence quantitative polymerase chain reaction, mRNA abundance of 29 target genes in peripheral blood mononuclear cells (PBMCs) were detected from 27 TAK patients and 10 healthy controls. Compared with the healthy control group, the untreated TAK group and the treated TAK group had an increased mRNA level of TLR2 and TLR4. A sample-to-sample matrix revealed that 80% of healthy controls could be separated from the TAK patients. Correlation analysis showed that the inactive-treated TAK group exhibited a unique pattern of inverse correlations between the TLRs gene clusters (including TLR1/2/4/6/8, BCL6, TIGIT, NR4A1, *etc*) and the gene cluster associated with T-cell activation and differentiation (including TCR, CD28, T-bet, GATA3, FOXP3, CCL5, *etc*). The dynamic gene co-expression network indicated the TAK groups had more active communication between TLRs and T-cell activation than healthy controls. BCL6, CCL5, FOXP3, GATA3, CD28, T-bet, TIGIT, IκBα, and NR4A1 were likely to have a close functional relation with TLRs at the inactive stage. The co-expression of TLR4 and TLR6 could serve as a biomarker of disease activity in treated TAK (the area under curve/sensitivity/specificity, 0.919/100%/90.9%). The largest gene co-expression cluster of the inactive-treated TAK group was associated with TLR signaling pathways, while the largest gene co-expression cluster of the active-treated TAK group was associated with the activation and differentiation of T-cells. The miRNA sequencing of the plasma exosomes combining miRDB, DIANA-TarBase, and miRTarBase databases suggested that the miR-548 family miR-584, miR-3613, and miR-335 might play an important role in the cross-talk between TLRs and T-cells at the inactive stage. This study found a novel relation between TLRs and T-cell in the pathogenesis of autoimmune diseases, proposed a new concept of TLR-co-expression signature which might distinguish different disease activity of TAK, and highlighted the miRNA of exosomes in TLR signaling pathway in TAK.

## Introduction

Toll-like receptors (TLRs) are a type of pattern recognition receptor that can initiate multiple immune responses by combining with pathogen-associated molecular patterns (PAMPs) and damage-associated molecular patterns (DAMPs). A growing number of studies have been demonstrated that TLRs take part in the pathogenesis of many kinds of autoimmune diseases (AIDs) through several mechanisms (1–3). Recently, NI-0101, an anti-toll-like receptor 4 monoclonal antibody was used in RA, which was the first clinical trial to target TLRs to treat autoimmune diseases (4). The dearth of knowledge on the role of TLRs in the pathogenesis of Takayasu’s arteritis (TAK) is especially notable when compared to the relatively higher number of studies on their role in that of rheumatoid arthritis (RA), systemic lupus erythematosus (SLE), and multiple sclerosis (MS).

TAK is a primary large vessel vasculitis mainly affecting the aorta and its major branches. Patients with onset TAK are typically female, and more than 90% of them are under 30 years old. Annual TAK incidence rates are estimated to be 1.5 cases per million in Japan, and 0.2~2.6 cases per million in Europe and North America (5). The autoimmune inflammation of TAK appears to be dominantly driven by T-cells (6). Studies have shown that there are numerous associations between TLRs and the activation and differentiation of T-cells. For instance, TLRs expressed in innate immune cells, such as DCs and macrophages, can regulate the activation and differentiation of T-cells. On the other hand, TLRs expressed in T-cells can influence T-cells more directly. For instance, TLR1 and TLR9 are highly expressed in CD4^+^ T cells, and CD8^+^ T cells have abundant TLR3 and TLR4 expression (7). TLR7-mediated suppression of Th17 cells does not require dendritic cell involvement (8), TLR2 signaling alters the transcriptional program of differentiating and increases the proliferation of Th17 cells (9), TLR8 signaling suppresses glucose uptake and metabolism in Treg cells (10), and TLR2 signaling enhances the movement of Treg cells (11), which all act on T-cell directly. Thus, we hypothesized that TLRs regulate the activation and differentiation of T-cells in TAK. However, there have been only two studies in the literature on TLRs in the pathogenesis of TAK in PubMed database (12, 13). Kabeerdoss et al. found that the higher mRNA expression of TLR4 and its ligand S100s in peripheral blood mononuclear cells (PBMCs) of TAK patients compared to healthy controls and that after being stimulated with TLR4 ligand (12), PBMCs from TAK patients had a higher mRNA expression of IL-1β and IL-1R2 compared to that of HC (13). Taken together, it has been currently unknown whether TLRs are related to the disease activity or the activation and differentiation of T-cells in the pathogenesis of TAK.

In organisms, genes form molecular networks, these molecular networks tend to be modular, and similar modules combine to function (14–16). If a network has a high clustering coefficient, it suggests the presence of local cliques or clusters of connected molecules (17). Genes with high expression similarity or linked by the shortest path are often involved in the same biological pathway or are subjected to shared regulatory pathways (18–21). The identification of stable and reliable gene co-expression networks is essential to unravel the interactions and functional correlations between genes (22). Analysis of the gene co-expression network is one of basic approaches currently adopted by research on the relations between two clusters of genes (14, 15).

Additionally, in this study, we defined ‘co-stimulatory molecules’ as both negative and positive co-stimulatory molecules (23). After T cell receptor (TCR) activation, the fate of the T cells is controlled by signals from T cell co-stimulatory molecules and cytokines largely.

The aim of this study was to determine whether a relation exists between TLRs and the activation and differentiation of T-cells in TAK and to analyze the key molecules that play an important role in this regulation. This study may provide experimental evidence for targeting TLRs in the treatment of TAK.

## Methods

**Figure 1:**
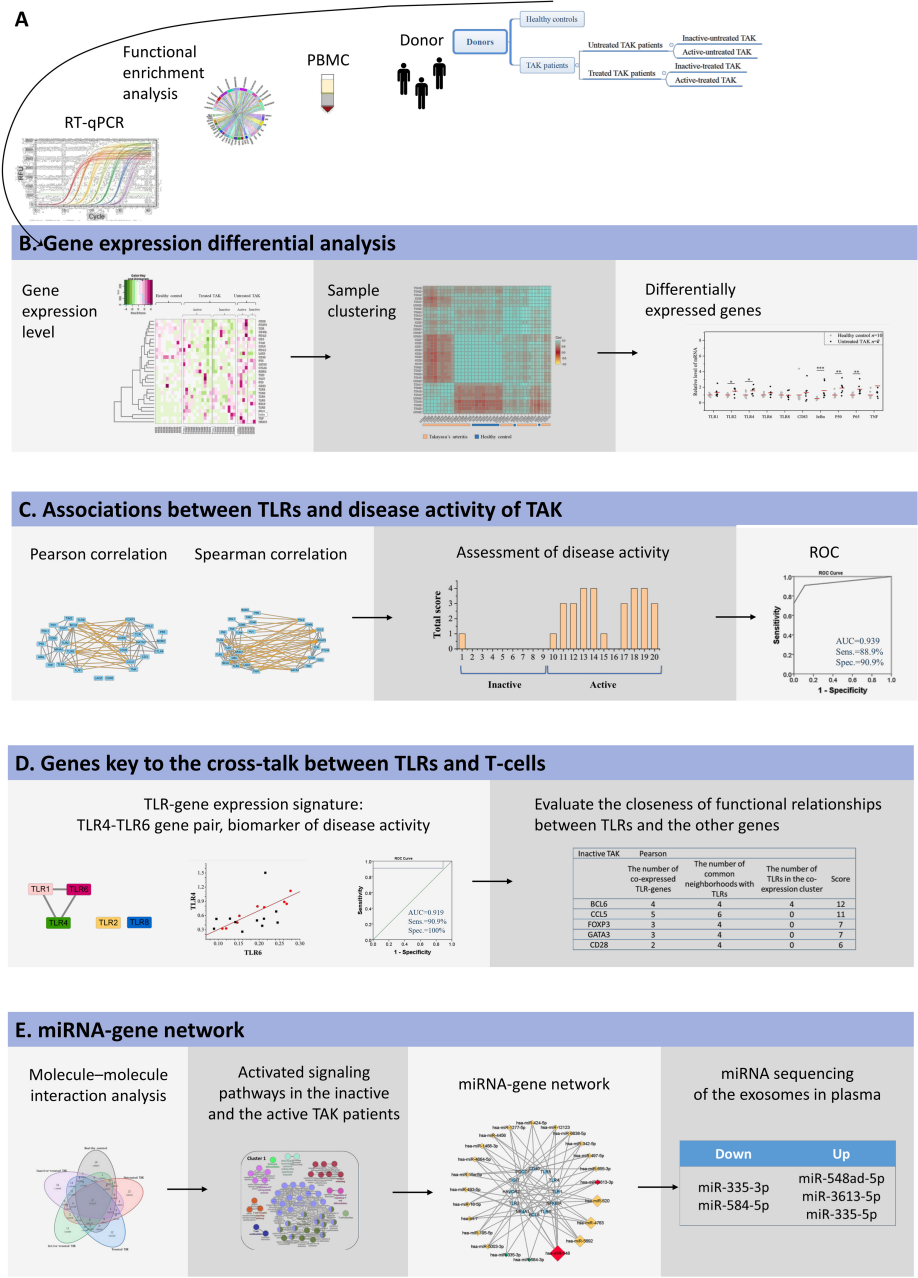
The basic workflow of this study. **(A)** mRNA abundance of 29 target genes in PBMCs was measured using RT-qPCR, and these genes were highly enriched in TLR signaling pathways and the function of the activation and differentiation of T-cells by functional enrichment analysis. **(B)** Sample clustering analysis suggested that TAK patients had a different expression pattern of the selected genes from the healthy controls. Gene expression differential analysis demonstrated the upregulation of TLR signaling pathway in TAK, but no substantial difference in TLR signaling pathway was found between the inactive-treated TAK patients and the active-treated TAK patients. **(C)** Correlation analysis showed that the inactive-treated TAK group exhibited a unique pattern of inverse correlations between the TLRs gene cluster and the gene cluster associated with T-cell activation and differentiation, while the active-treated TAK group did not. And TLRs and their correlation cluster could distinguish active patients from inactive patients in TAK. **(D)** Dynamic gene co-expression network was constructed. TLR-co-expression signature of different stages was observed, and the degree of functional association between the other genes with TLRs was assessed. **(E)** To account for the co-expression in the greatest cluster of the inactive-treated TAK group, functional enrichment analysis, miRNA database prediction, and miRNA sequencing of plasma exosomes was performed, which indicated that miRNAs might play an important role in the cross-talk between TLR and T-cell in TAK patients.

**Figure 1** Summarized the basic workflow of this study.

### Gene Function Annotation

Universal Protein Resource (UniProt) SwissProt database was used to annotate gene functions (24).

### Functional Enrichment Analysis

Functional enrichment analysis was performed by the Metascape webserver (25) (https://metascape.org/), and the pathway databases consisted of Gene Ontology (GO) (26), KEGG (27), and WikiPathways (28). Enrichment analysis was performed by using a hypergeometric test with a *p*-value cutoff of 0.01. The enrichment results were visualized using the R Package ggplot2, the R Package Circlize, and Cytoscape V. 3.8.2.

### Networks Based on Public Databases

The gene co-expression network was constructed using the COEXPEDIA database (29) (www.coexpedia.org), and the protein-protein interaction (PPI) networks were constructed using the STRING database (30) (https://www.string-db.org/).

### Patients

Treated TAK patients fulfilling the 1990 ACR criteria (31) were enrolled. And we assessed the disease activity of TAK by the 1994 NIH criteria (32), which included the following.

1. Systemic features, such as fever, musculoskeletal (no other cause identified).
2. Elevated ESR.
3. New onset or aggravated features of vascular ischemia or inflammation, such as claudication, diminished or absent pulse, bruit, vascular pain (carotodynia), asymmetric blood pressure in either upper or lower limbs (or both).
4. Typical angiographic features.

 If a TAK patient had two or more features, he was defined as “active TAK patient”; otherwise, we diagnosed the patient was at remission stage, and the patient was defined as “inactive TAK patient”.

A patient that never received any TAK medication was defined as “untreated TAK patient”, and a patient that was under treatment was defined as “treated TAK patient”. Both the untreated and the treated patients were classified into the inactive group and the active group.

Written informed consent was obtained from all participants and the study was performed in accordance with the Declaration of Helsinki. And this study was approved by the Institutional Review Board of Peking Union Medical College Hospital, Beijing, China (S-478).

### Collection and Processing of Human Blood Samples

PBMCs were isolated from patients by density-gradient centrifugation. Total RNA was prepared from the PBMCs using Trizol reagent (15596026, Thermo Fisher Scientific) (33). The RNA samples were diluted in RNase-free water, denatured at 65°C for 10 min. RNA concentration and purity were determined spectrophotometrically, and the RNA integrity was verified by denaturing RNA gel electrophoresis.

### Real-Time Fluorescence Quantitative Polymerase Chain Reaction (RT-qPCR)

RNA was reverse transcribed using the PrimeScript™ RT reagent Kit with gDNA Eraser (RR047A, Takara). Genomic DNA (gDNA) was eliminated at 42°C for 2 min. Reverse transcription was performed using the following conditions: 37 °C for 15 min, 85 °C for 5 sec. RT-qPCR reactions were performed with the iTaqTM Universal SYBR^®^ Green Supermix (725124, Bio-Rad) and primers were listed in **Supplementary Table 1**. The temperature cycle parameters in an Applied Biosystem 7900HT1 were: 95°C for 30 sec and 40 cycles of 95°C for 30 sec, 56°C for 30 sec and 72°C for 40 sec followed by a hold at 72°C for 40 sec. Gene expression was calculated using the 2^−ΔΔCq^ method. Melting curve analysis was performed from 65 to 95 °C.

### The Targeted Genes

Total 29 genes were selected which were confirmed closely related to TLR signaling pathways and the function of the activation and differentiation of T-cells by GO and KEGG enrichment analysis, including BCL6, CCL5, CD28, CD3 (CD247), CD40, CD40L (CD40LG), CD83, CTLA4, FOXP3, GATA3, IκBα (NFKBIA), LAG3, NR4A1, P50 (NFKB1), P65 (RELA), PD-1 (PDCD1), PD-L1 (CD274), PD-L2 (PDCD1LG2), RORC, T-bet (TBX21), TCR (TRA), TIGIT, TIM3 (HAVCR2), TLR1, TLR2, TLR4, TLR6, TLR8, and TNF.

### The Selection of Reference Genes

A list of 9 genes previously reported that were stably expressed in human PBMCs from the literature was compiled, including ACTB, β-glucuronidase, B2M, GAPDH, HPRT1, PGK1, RPL13A, SDHA, and YWHAZ. We Assessed the gene expression stability and selected the most appropriate housekeeping genes for each analysis using the geNorm (34), the NormFinder (35), and the BestKeeper software (36). As a result, B2M and YWHAZ were used as the internal reference genes for the comparison of healthy controls and untreated TAK patients, B2M and SDHA were used for the comparison of healthy controls and treated TAK patients and the comparison of the active-treated and the inactive-treated TAK patients, and HPRT1 and YWHAZ were used for the comparison of the untreated and the treated TAK patients and correlation analysis.

### Dynamic Gene Co-Expression Network

We constructed the gene co-expression network of the healthy control group, the untreated TAK group, the treated TAK group, the inactive-treated TAK group, and the active-treated TAK group based on the qPCR dataset, respectively. The Pearson correlation has an advantage in predicting the interaction of molecules and is widely used as a measure of gene co-expression in many public databases (37, 38), so it was adopted the main approach in the network analysis and the results were available in the main text. However, Spearman correlation has an advantage in revealing the functional associations, so it was adopted as a complementary approach and the results are available in the **Supplementary Information** (39). Statistical analysis was performed using IBM SPSS statistic V.23 (Armonk, New York, USA). A *p*-value of less than 0.05 was considered significant. Cytoscape V. 3.8.2 was used to edit the networks.

### Unsupervised Hierarchical Clustering Analysis and the Co-Expression Cluster

Samples were clustered based on the Pearson correlation coefficient for the profile of those 29 genes using the R package ggcorrplot. Hierarchical clustering of samples was carried out using Pearson correlation and Spearman correlation respectively (38). Data were partitioned into five clusters by cutting the clustering tree at the height of 1.0 and the co-expression clusters were defined which consist of similarly expressed genes. The clustered heatmap and hierarchical clustering trees were performed using the R package ggcorrplot and the R package ggplot2 by R V.4.1.0. A *p*-value of less than 0.05 was considered significant.

### Scoring System for the Assessment of Disease Activity

The disease activity score was calculated using the algorithm as published before (40). To assess the disease activity of TAK, highly co-expressed gene pairs (defined as |r| > 0.73, p < 0.01) of the inactive-treated were selected. Regression equations were calculated by linear least squares. To qualify how well the data of an individual sample fit a regression line, we calculated the relative error (RE) as the ratio of the absolute error (AE) and the observed value of gene expression level. The AE is the absolute value of the difference between the predictive value and the observed value.

M-value was introduced which was equal to the RE:

$M=\left|\;\left(y_{predictive\;value}-y_{observation}\right)/y_{observation}\right|.$ (40)

Qualified the data of each sample to fit every regression line one by one. The *M*-value of each active or inactive patient was calculated. Then the patients were ranked by *M*-value. The threshold was set using the Youden index and was adjusted when appropriate. A score of 1 was given if a patient had an *M*-value less than the threshold, 0 if a patient had an *M*-value greater than the threshold. The total score of a patient was the sum of each score of *M-*value. The threshold of the total score was set using the Youden index. Detailed protocols are available in ref.

### Evaluate the Closeness of Gene-to-Gene Functional Relationships Between TLRs and the Other Genes

To predict the functional relationship between TLRs and the other genes through quantification, the following metrics were employed, and its rationale has been published (19–21, 41–43). Take BCL6 for an example.

1. The number of TLR-genes that were co-expressed with BCL6.
2. The number of common neighborhoods with TLRs. In the metric, all TLRs were processed as a whole-body corresponding to one gene.
3. The number of TLRs in the co-expression cluster that consisting of BCL6, which cluster was cut at the height of 1.0.

One gene of each metric was counted as one point. Calculated the total score of each gene assessed.

### miRNA-Gene Network Prediction

The pipeline was composed of three major steps:

1. miRNAs targeting each gene were obtained from the miRDB database, which is a miRNA target prediction database (44, 45).
2. Counted the number of genes of each miRNA listed in (i), and the miRNA which targets more than one gene was selected.
3. Mapping according to the miRNA-gene pair list acquired in (ii). In this step, the miRNAs were pooled for miRNA families if there were more than one miRNA belonging to the same miRNA family.
4. Ranked the miRNAs or the miRNA family according to the number of genes targeted.

### Plasma Preparation

Whole blood was collected from controls and TAK patients in ethylenediaminetetraacetic acid (EDTA) tubes and stored at 4°C. Plasma was isolated immediately from the whole blood by centrifugation (4°C, 800 × g for 10 min) in 4 hours. Then, the collected plasma was isolated by further centrifugation (4°C, 3,000 × g for 15 min) to remove cellular debris and large vesicles. Plasma samples were stored at −80°C until analysis.

### Exosome Isolation and Quantification for miRNA Sequencing

Plasma samples were thawed at 37°C and filtered through a filtration membrane (0.8 μm). Next, the plasma was diluted 1:1.5 in filtered 0.01 M phosphate-buffered saline (PBS). Exosomes were purified by consecutive steps of size exclusion chromatography (SEC) on size exclusion columns and ultrafiltration tubes (100 kDa cutoff, Amicon). Successful exosome isolation was confirmed as follows.

1. Immunoblotting analysis revealed negative calnexin (Proteintech), positive CD63 (SanTAK-Cruz), positive CD9 (Proteintech), and positive TSG101(Absin).
2. Immuno-electron microscope analysis and nanoparticle tracking analysis (NTA) revealed that the isolated exosomes were 30~150 nM in diameter.

RNA was isolated using miRNeasy Mini Kit (Qiagen) from exosomes. All the RNA samples met the following RNA quality threshold.

1. The ratio of OD260/280 was between 1.8 and 2.0.
2. The RNA concentration was greater than 300 pg/μL.
3. The Cq value of miRNA-130b was less than or equal to 23.
4. 16S rRNA (-).

### miRNA Sequencing

Small RNA libraries were prepared with the QIAseq miRNA Library Kit (QIAGEN). The quality of the libraries was validated on an Agilent Bioanalyzer 2100 and qPCR. Pair-end sequencing was performed on Illumina HiSeq2500.

### Statistical Analysis

Normality was assessed with a Kolmogorov–Smirnov. Normally distributed continuous variables were provided as mean ± standard deviation and non-normally distributed continuous variables as median (interquartile). A Chi-square test was used for reporting associations between two categorical variables. Differences of continuous variables between groups were analyzed by the Mann–Whitney test. The correlation between gene expression levels was represented by the Pearson correlation coefficient. The models were otherwise validated by examining standardized residuals for normal distribution. Statistical analysis was performed using IBM SPSS statistic V.23 (Armonk, New York, USA). A *p*-value of less than 0.05 was considered significant.

## Results

### Demographic Data, Clinical Features, and Laboratory Findings of Patients

Twenty-seven TAK patients were included. Based on the definition described above, 7 patients were classified into the untreated TAK group, and 20 patients were classified into the treated TAK group. Among them, 15 patients were assessed for remission, and 12 were assessed as being at the active stage following the NIH criteria described previously. Besides, 10 healthy subjects served as controls.

The demographic data and laboratory findings of patients were listed in **Supplementary Table 2**, and the individual patient data were seen in **Table 1**. There was no significant difference between the inactive-treated TAK group and the active-treated TAK group in age (*p*=0.82), sex (*p*=0.35), disease duration (*p*=0.1), erythrocyte sedimentation rate (ESR) (*p*=0.33), IL-6 (*p*=0.1), tumor necrosis factor (TNF)-α (*p*=0.66), and the dose of corticosteroid (*p*=0.37). The active-treated TAK group had a higher hypersensitive-C reactive protein (hs-CRP) level than the inactive-treated TAK group (*p*=0.02).

**Table 1:** Demographic data and clinical features of patients with Takayasu’s arteritis.

	Age (years)	Gender	Disease duration (months)	ESR (mm/h)	hs-CRP (mg/L)		Interleukin 6 (pg/mL)	TNF-α (pg/mL)	Prednisone
				(ref. range, 0~20)	(ref. range, 0~8.00)		(ref. range, <5.9)	(ref. range, <8.1)	(mg/d)
**Treated TAK patients (*n*=20)**									
*Inactive (n=11)*									
# 1	32	F	3	5	1	4.4		8.8	24
# 2	51	F	12	21	2.86	3		7.5	10
# 3	39	F	88	11	2.92	2		6.4	0
# 4	34	F	51	12	0.84	2		4	5
# 5	49	F	34	9	0.17	2		6	7.5
# 6	28	F	40	7	0.91	2		6.1	7.5
# 7	26	F	5	17	0.31	2		11	45
# 8	59	F	43	12	0.44	2		4	35
# 9	37	F	44	13	0.21	2.4		5.6	10
*Active (n=9)*									
# 10	36	F	133	16	0.77	2		7.8	40
# 11	32	F	132	33	5.4	5.8		6.1	10
# 12	50	F	380	19	14.7	5.7		8	15
# 13	40	F	11	38	7.8	9.3		7.6	15
# 14	25	F	19	16	23.7	7.5		4	10
# 15	38	F	118	12	3.64	–		–	10
# 16	34	F	58	6	0.34	2		5.2	45
# 17	39	F	13	23	–	2.1		5.9	44
# 18	49	F	7	5	0.55	2.8		9.5	10
# 19	40	F	314	1	5.85	3.5		24.5	10
# 20	50	M	200	16	8.51	2		5.6	0
**Untreated TAK patients (*n*=7)**									
*Inactive (n=3)*									
# 21	34	F	176	7	0.34	2		4.3	0
# 22	27	F	5	14	0.16	25.7		4	0
# 23	38	F	48	5	0.32	3		4	0
*Active (n=4)*									
# 24	31	M	1	91	140.72	–		–	0
# 25	25	F	1	19	11.28	6.3		5.2	0
# 26	23	M	81	71	77.36	6.3		6.2	0
# 27	29	F	4	127	113.62	22.2		8.4	0

M, male; F, female; ESR, erythrocyte sedimentation rate; hs-CRP, hypersensitive- C reactive protein; TNF-α, Tumor Necrosis Factor-α; ref., reference.

### Functional Enrichment Analysis and Interaction Analysis of Selected Genes

**Figure 2:**
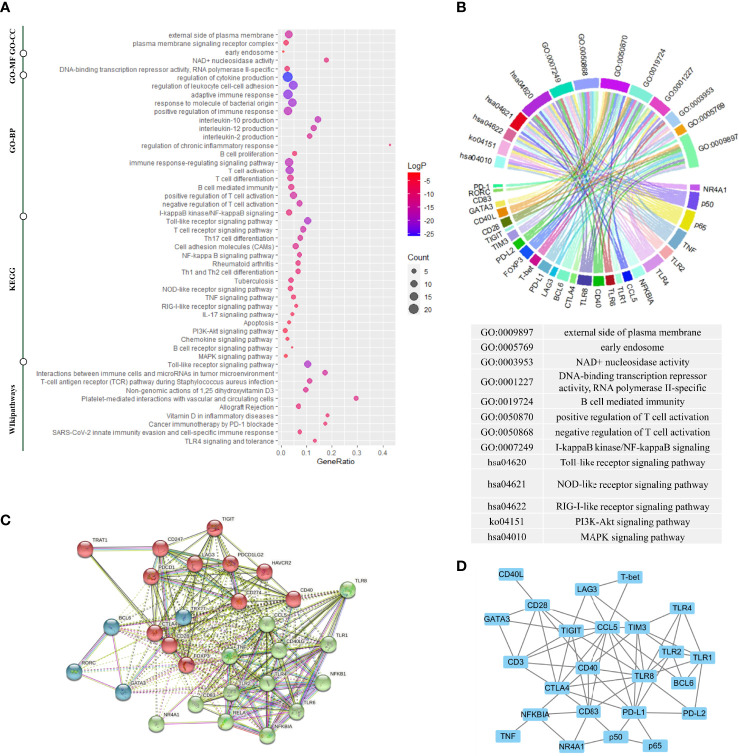
Gene enrichment analysis, protein–protein interaction analysis, and gene co-expression analysis. **(A)** Gene enrichment analysis using multiple databases including the Kyoto Encyclopedia of Genes and Genomes (KEGG) database, the Gene Ontology (GO) database, and the WikiPathways database for targeted genes. **(B)** The genes corresponding to the enrichment results of GO analysis and KEGG analysis. **(C)** The protein–protein interaction (PPI) analysis based on the STRING database. **(D)** The gene co-expression analysis based on the COEXPEDIA database. Functional enrichment analysis indicated these genes were closely related to TLR signaling pathways and the function of the activation and differentiation of T-cells. PPI analysis and gene co-expression analysis indicated that there were strong associations among these genes, and experimental co-expression relationships would be compared with these database-interactions for newly discovered co-expression relationships.

The function of the selected genes according to UniProt was summarized in **Supplementary Table 3**. To gain further insight into the function of the 29 selected genes, we performed a functional enrichment analysis based on GO, KEGG, and WikiPathways databases. The functional enrichment analysis results confirmed that the 29 selected genes were closely related to this research topic. **Figure 2A** showed these genes were highly enriched in TLR signaling pathways and the function of the activation and differentiation of T-cells. **Figure 2B** showed the one-to-one correspondence between the genes and some GO terms and KEGG pathways which were closely related to this research topic, including positive/negative regulation of T-cell activation, I-kappaB kinase/NF-kappaB signaling, and Toll-like receptor signaling pathway., and other functions or pathways that are crucial to the pathogenesis of AIDs, such as B-cell mediated immunity, regulation of interleukin production, external side of plasma membrane, NAD+ nucleosidase activity, DNA-binding transcription repressor activity, RNA polymerase II-specific, NOD-like receptor signaling pathway, RIG-I-like receptor signaling pathway, PI3K-Akt signaling pathway, and MAPK signaling pathway. The detailed enrichment results were seen in **Supplementary Table 4**.

Next, to characterize possible molecular interactions across these selected genes, we built a protein-protein interaction (PPI) map using the STRING database. **Figure 2C** showed the PPI network clustering-based K-means (k = 3). The 3 clusters were each labeled with a different color, in which the proteins were functionally related. The genes labeled as red coded the first signaling molecule of T-cell activation or co-stimulatory molecules except FOXP3, the genes labeled as blue coded key subset transcription factors, and most genes labeled as green belonged to TLR signaling pathway. Notably, NR4A1 was in the same cluster as RELA, NFKB1 and, NFKBIA. In addition to PPI networks, we also identified gene interaction using gene co-expression network (16). **Figure 2D** showed the gene co-expression network based on Coexpedia database, indicating the strong functional association among these genes. And these interactions would align with our experimental results later.

### Sample Clustering Analysis and Gene Expression Differential Analysis in TLR Signaling Pathway

**Figure 3:**
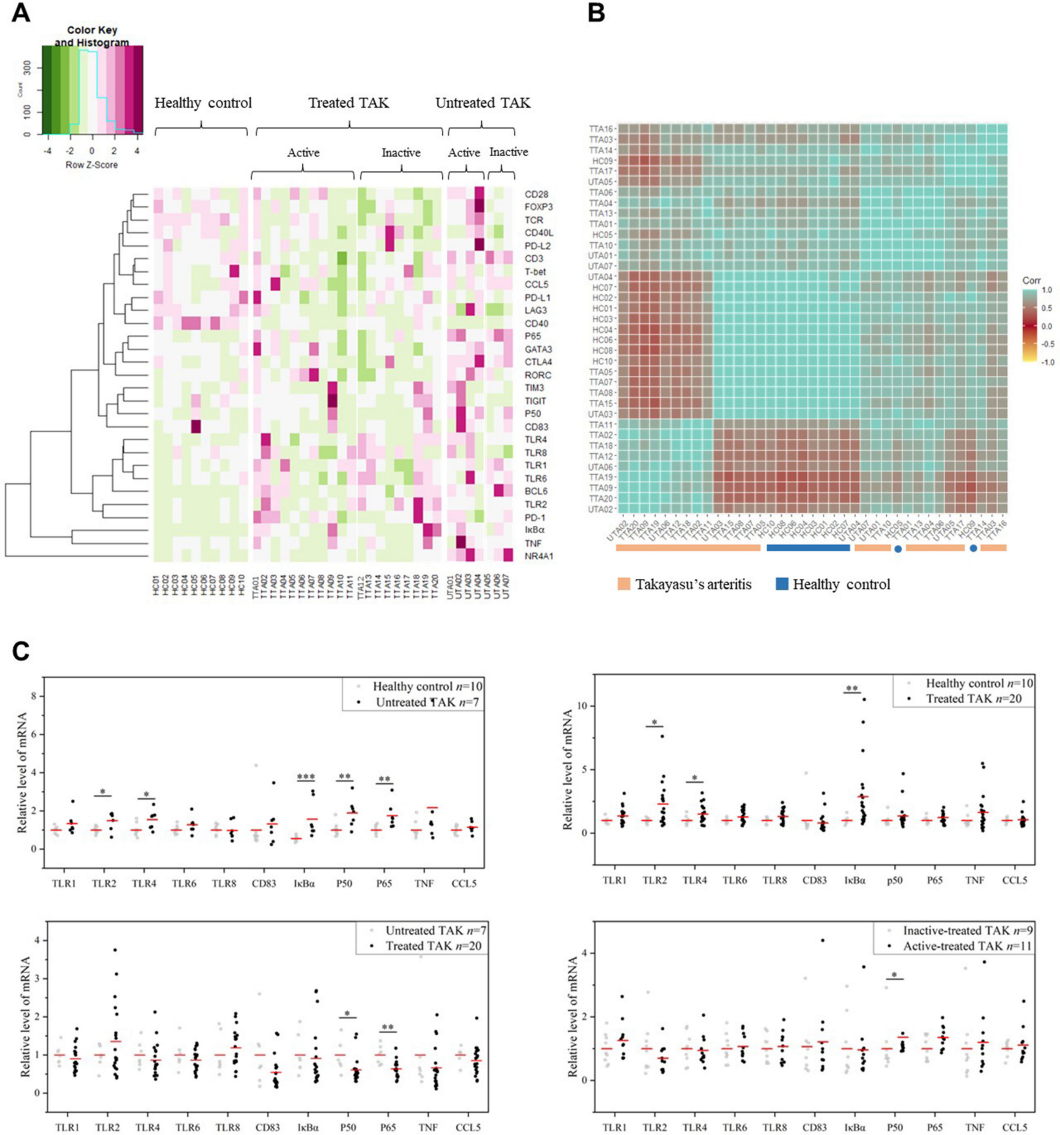
The gene expression levels. **(A)** The heatmap showing gene expression levels with corresponding dendrograms using hierarchical clustering (Euclidean distance measure). **(B)** The heatmap of sample clustering using hierarchical clustering (Pearson correlation), suggesting that TAK patients had a different expression pattern of the selected genes from the healthy controls. **(C)** Gene expression differential analysis of TLR signaling pathway. Mann–Whitney test. *p < 0.05. **p < 0.01, ***p < 0.001. The red center line represented the mean value of the mRNA level. TAK, Takayasu’s arteritis.

The heat map displayed the expression levels of the selected genes in individual samples, with red color indicating greater expression level, which showed a distinct pattern of gene expression in TAK patients compared with healthy controls, in untreated patients compared with treated patients, and in inactive patients compared with active patients (**Figure 3A**). Next, to identify the power of the selected genes to distinguish different sample groups, we performed the unsupervised hierarchical clustering of different samples based on the gene expression level calculated by Pearson correlation coefficients. A sample-to-sample matrix revealed that 80% (8/10) of healthy controls could be separated from the TAK patients by hierarchical cluster analysis, which suggested that TAK patients had a different expression pattern of the selected genes from the healthy controls (**Figure 3B**).

Among the selected genes, TLR1, TLR2, TLR4, TLR6, TLR8, CD83, IκBα, p50, p65, TNF, and CCL5 were key genes in TLR signaling pathway. As gene expression differential analysis showed, compared with the HC, the untreated TAK patients had higher mRNA levels of TLR2 (*p*=0.043), TLR4 (*p*=0.014), IκBα (*p*=0.00021), p50 (*p*=0.00046), and p65 (*p*=0.00031), and the treated TAK patients had higher mRNA levels of TLR2 (*p*=0.015), TLR4 (*p*=0.044), and IκBα (*p*=0.00016). Compared with the untreated TAK patients, the treated TAK patients had lower mRNA levels of p50 (*p*=0.013), and p65 (*p*=0.021). Compared the active-treated TAK patients with the inactive-treated TAK patients, p50 mRNA level (*p*=0.038) was higher and no increase in the mRNA level of TLRs was observed. (**Figure 3C**). Although there was evidence supporting the upregulation of TLR signaling pathway in TAK, no substantial differences in TLR signaling pathway were found between the inactive-treated TAK patients and the active-treated TAK patients.

### TLRs and Their Correlation Cluster Distinguish Active Patients From Inactive Patients in TAK

**Figure 4:**
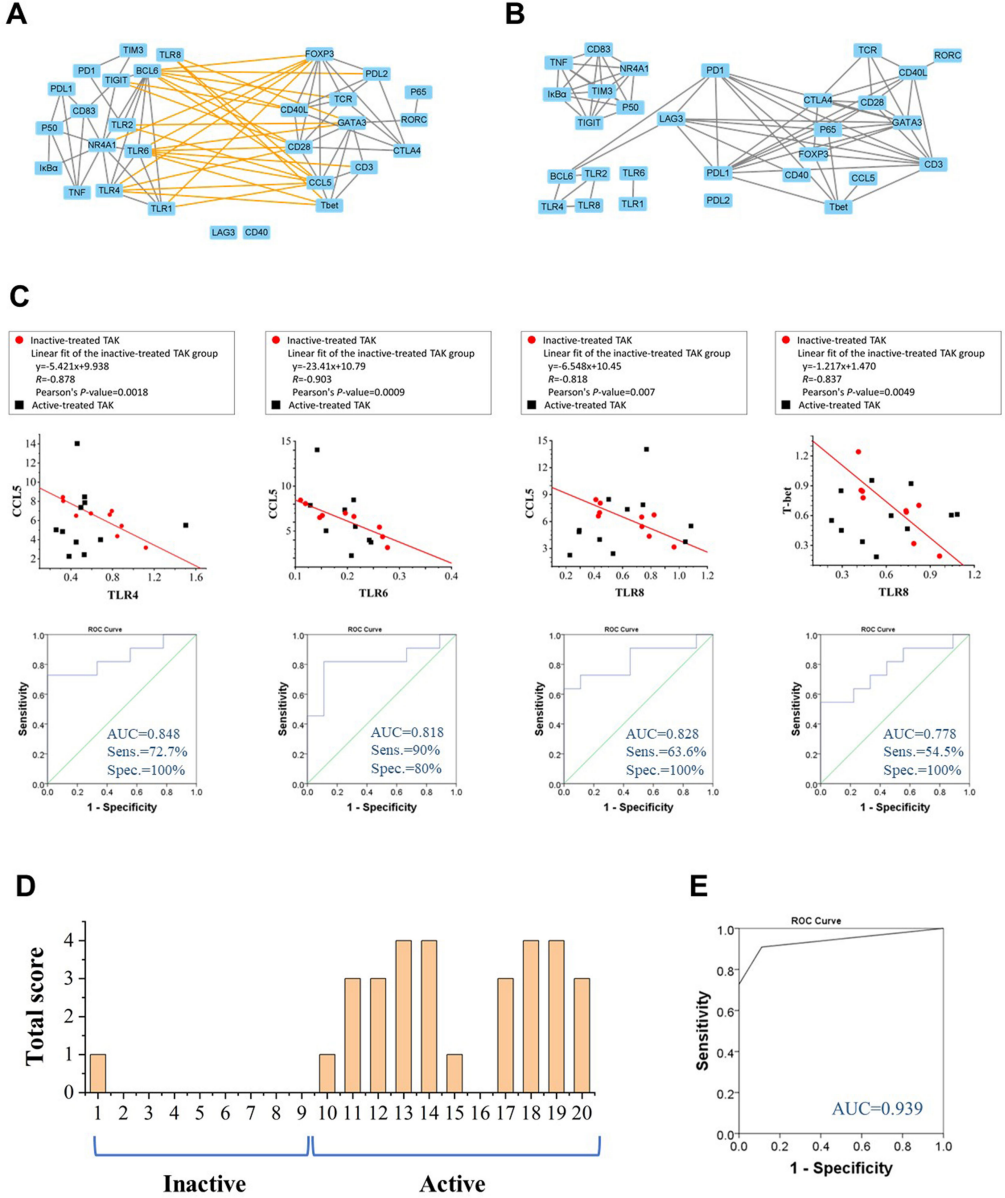
Toll-like receptors (TLRs) and their correlation cluster distinguish active patients from inactive patients in TAK. **(A, B)** The Pearson correlation between the expression levels of each gene pair of the inactive-treated TAK group **(A)** and the active-treated TAK group **(B)** showed that the inactive-treated TAK group exhibited a unique pattern of inverse correlations between the TLRs gene cluster and the gene cluster associated with T-cell activation and differentiation, while the active-treated TAK group did not. **(C)** The scatter plots, the linear regression, and the receiver operating characteristic curve (ROC) of the TLR4-CCL5 pair, the TLR6-CCL5 pair, the TLR8-CCL5 pair, and the TLR8-Tbet pair. **(D, E)** The ROC analysis was used to evaluate the assessment accuracy for the disease activity of TAK. The total score of each treated-TAK patient **(D)** and the ROC **(E)** when assessing the disease activity using the summed scoring system consisting of the above 4 gene pairs. TAK, Takayasu’s arteritis.

To more rigorously evaluate the functional relations among these genes, we calculated the pairwise Pearson correlation and the pairwise Spearman correlation between each pair of genes. Although Pearson correlation is better suited for the establishment regression equations, we adopted Spearman correlation as a complementary analysis as Spearman correlation has some advantages in reflecting the functional associations between genes (39). Due to space limitations, most of the results based on Spearman correlation were provided in the supplementary materials, and Pearson correlation coefficient was presented in the text and adopted in the subsequent mathematical modeling. The most striking results to emerge from the data was that the inactive-treated TAK group exhibited a unique pattern of inverse correlations between the TLRs gene cluster (including TLR1/2/4/6/8, BCL6, TIGIT, NR4A1, IκBα, p50, TNF, CD83, PD-1, PD-L1, and TIM3), and the gene cluster associated with T-cell activation and differentiation (including TCR, CD28, T-bet, GATA3, FOXP3, CCL5, CD3, CD40L, CTLA4, PD-L2), either performed by Pearson correlation (**Figure 4A**) or Spearman correlation analysis (**Supplementary Figure 1A**) analysis, while the active-treated TAK group did not (**Figure 4B** and **Supplementary Figure 1B**). To more clearly demonstrate this feature, **Supplementary Figures 1C–F** only showed the high correlations (defined as |*r|* > 0.73, *p* < 0.01).

CCL5 is a potent chemoattractant for blood monocytes, memory T-helper cells, and eosinophils, which is important in recruiting T-cells into inflammatory sites, and also activates the apoptotic cell death pathway in T cells (46). Among these inverse correlations, CCL5 was negatively co-expressed with TLR1 (Pearson’s *r*= -0.675, *p*= 0.046), TLR2, TLR4 (Pearson’s *r*= -0.878, *p*= 0.002), TLR6 (Pearson’s *r*= -0.903, *p*= 0.001), and TLR8 (Pearson’s *r*= -0.818, *p*= 0.007), and T-bet was negatively co-expressed with TLR4 (Pearson’s *r*= -0.713, *p*= 0.031), TLR6 (Pearson’s *r*= -0.755, *p*= 0.019), and TLR8 (Pearson’s *r*= -0.837, *p*= 0.005). We took the TLR4-CCL5 pair, the TLR6-CCL5 pair, the TLR8-CCL5 pair, and the TLR8-T-bet pair to establish linear regression models to assess the disease activity, and the values of AUC/sensitivity/specificity were 0.848/72.7%/100%, 0.818/63.6%/100%, 0.828/90%/80%, and 0.778/54.5%/100%, respectively (**Figure 4C**). We then calculated the total score from the above 4 gene-pair models for each patient to assess the disease activity as mentioned in *Method*, the AUC, the sensitivity, the specificity, the positive predictive value, and the negative predictive value were 0.939, 90.9%, 88.9%, 90.9%, and 88.9%, respectively, which had greater diagnostic accuracy than did the single models (**Figures 4D, E**). The assessment of individual patients was shown in **Supplementary Table 5**. The above results suggest that TLRs have a potential relationship with the disease activity of TAK, which requires further explanation.

### Topological Analysis of Dynamic Gene Co-Expression Network Identifies the Functional Association Between TLRs and T-Cell Activation in TAK

#### The More Active Communication Between TLRs and T-Cell Activation in TAK Compared to Healthy Control

**Figure 5:**
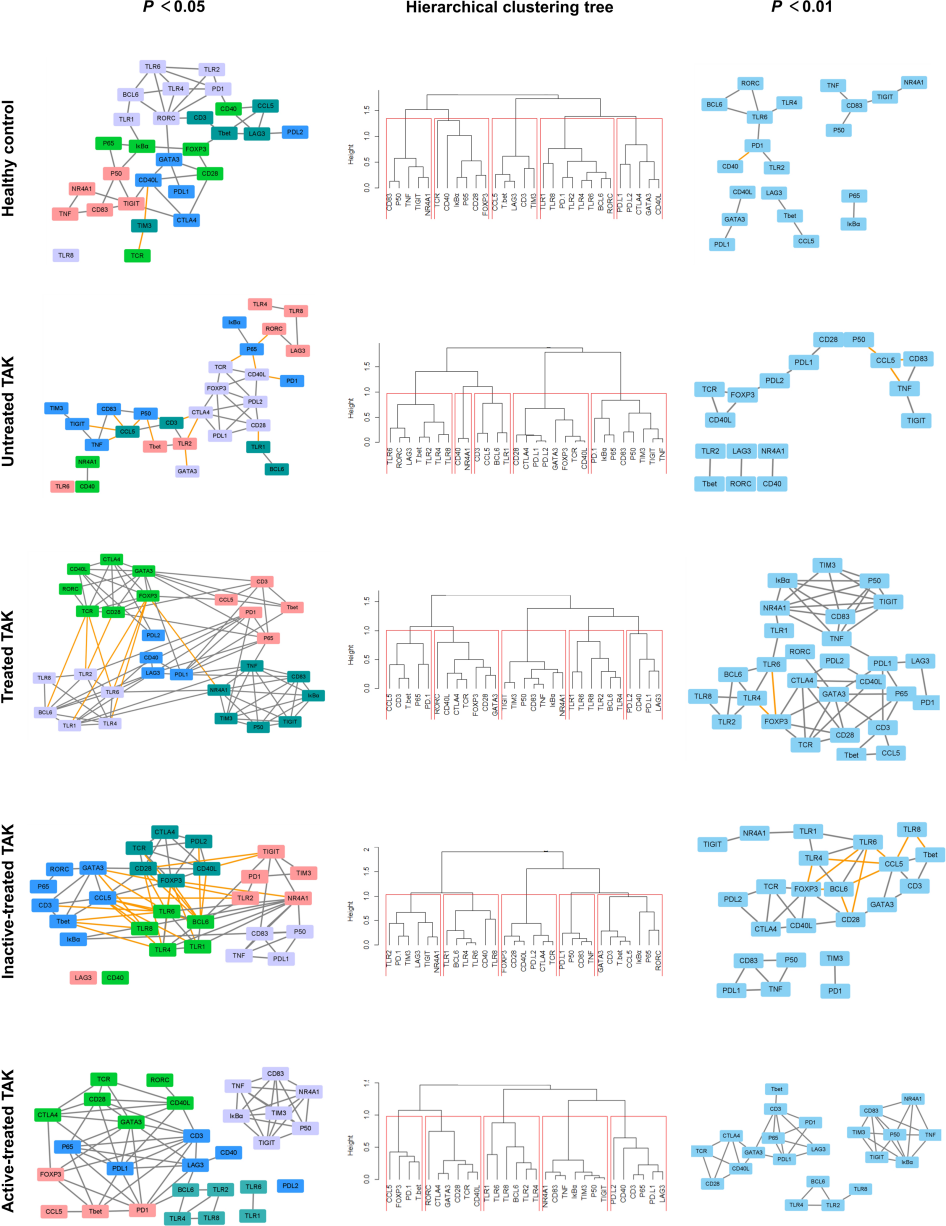
Dynamic gene co-expression networks based on Pearson correlation. Left panel, the gene co-expression networks consisting of correlation with a *p*-value less than 0.05. Right panel, to more clearly demonstrate this feature, only the high correlations (defined as |*r|* > 0.73, *p* < 0.01) were shown. Middle panel, the hierarchical clustering trees using complete method. As a result, genes were organized into 5 clusters by cutting the clustering tree at the height of 1.0, which was indicated by red frames. Genes belonging to the same cluster were like to have similar functions and were labeled with the same color. The negative correlation was indicated by the yellow edge, while the positive correlation was gray in the networks.

Gene co-expression network analysis is a useful method to link tightly co-expressed gene modules to phenotypic traits (47). To determine gene modules associated with distinct disease stages of TAK, we constructed a dynamic gene co-expression network for each group based on the pairwise Pearson correlations or Spearman correlations for all genes. (**Figure 5** and **Supplementary Figure 2**). Unsupervised hierarchical clustering was used to assess the function of genes. As a result, genes were organized into 5 clusters by cutting the clustering tree at the height of 1.0, which was indicated by red frames. Notably, genes belonging to the same cluster were like to have similar functions and were labeled with the same color. In active-treated TAK, the 5 clusters were as follows.

1. Cluster No. 1 (cutting the clustering tree at the height of 2.0)
  - TLR1, TLR2, TLR4, TLR6, TLR8, and BCL6.
  - CD83, TNF, NR4A1, IκBα, p50, NR4A1, TIM3, and TIGIT.
  - CD40, PD-L1, PD-L2, LAG3, CD3, and p65.
2. Cluster No. 2 (cutting the clustering tree at the height of 2.0)
  - TCR, CD28, CD40L, CTLA4, GATA3, and RORC.
  - T-bet, CCL5, FOXP3, and PD-1

In inactive-treated TAK, the 5 clusters were as follows.

1. Cluster No. 1 (cutting the clustering tree at the height of 2.0)
  - TLR1, TLR4, TLR6, BCL6, and CD40.
  - TLR2, PD-1, TIM3, LAG3, TIGIT, and NR4A1.
2. Cluster No. 2 (cutting the clustering tree at the height of 2.0)
  - TCR, CD28, CD40L, CTLA4, FOXP3, and PD-L2.
  - CD3, CCL5, T-bet, GATA3, RORC, IκBα, and p65.
  - PD-L1, p50, CD83, and TNF.

Comparing the two results, it could be seen that the 29 genes were divided into two broad categories: (i) TLR signaling pathway and (ii) the activation and differentiation of T-cells. But compared with the inactive group, CD83, TNF, IκBα, p50, p65 as well as the co-stimulatory molecules (including PD-L1, PD-L2, and LAG3) were more closely related to TLRs in the active group. Besides, to present the main framework of the network clearer, another dynamic network with the observed value of the Pearson correlation was 0.01 was shown in the right column of **Figure 5** and **Supplementary Figure 2**. This co-expression clustering revealed a functional association among the genes, providing insight into gene functions and networks.

To assess the functional communication among these genes at distinct stages, we calculated a number of topological network parameters commonly used to describe the network. Each of these networks had a short characteristic path length and a large clustering coefficient, suggesting that they participate in the biological processes that might be functionally related. Additionally, compared with the healthy controls, the active-treated TAK and the inactive-treated TAK showed shorter characteristic path length (healthy controls vs. active-treated TAK vs. inactive-treated TAK, Pearson correlation 3.265 vs. 2.362 vs. 2.333, Spearman correlation 4.410 vs. 2.439 vs. 3.392) and a larger clustering coefficient (Pearson correlation 0.317 vs. 0.581 vs. 0.549, Spearman correlation 0.432 vs.0.489 vs. 0.470), indicating the more active functional communication among these genes in TAK groups compared to healthy controls. The detailed parameters of these networks are shown in **Table 2**.

**Table 2:** Parameters of gene co-expression networks.

	Healthy control (n = 10)	Untreated patients with TAK (n = 7)	Treated patients with TAK (n = 20)	Treated patients with inactive TAK (n = 9)	Treated patients with active TAK (n = 11)
***Pearson correlation***					
Nodes	29	29	29	29	29
Edges	46	42	95	79	72
Average number of neighbors	3.286	3.154	6.552	5.852	5.263
Network diameter	7	11	4	6	6
Clustering coefficient	0.317	0.285	0.632	0.581	0.549
Characteristic path length	3.265	4.025	2.155	2.362	2.333
***Spearman correlation***					
Nodes	29	29	29	29	29
Edges	43	22	114	89	66
Average number of neighbors	3.071	2.222	8.143	6.357	4.714
Network diameter	11	9	4	6	9
Clustering coefficient	0.432	0.213	0.597	0.489	0.470
Characteristic path length	4.410	4.144	1.910	2.439	3.392

TAK, Takayasu’s arteritis.

#### TLR-Co-Expression Signature: The Regression Equation Relating the TLR6 mRNA Level to the TLR4 mRNA Level Serves as a Biomarker of Active Disease in Treated TAK

**Figure 6:**
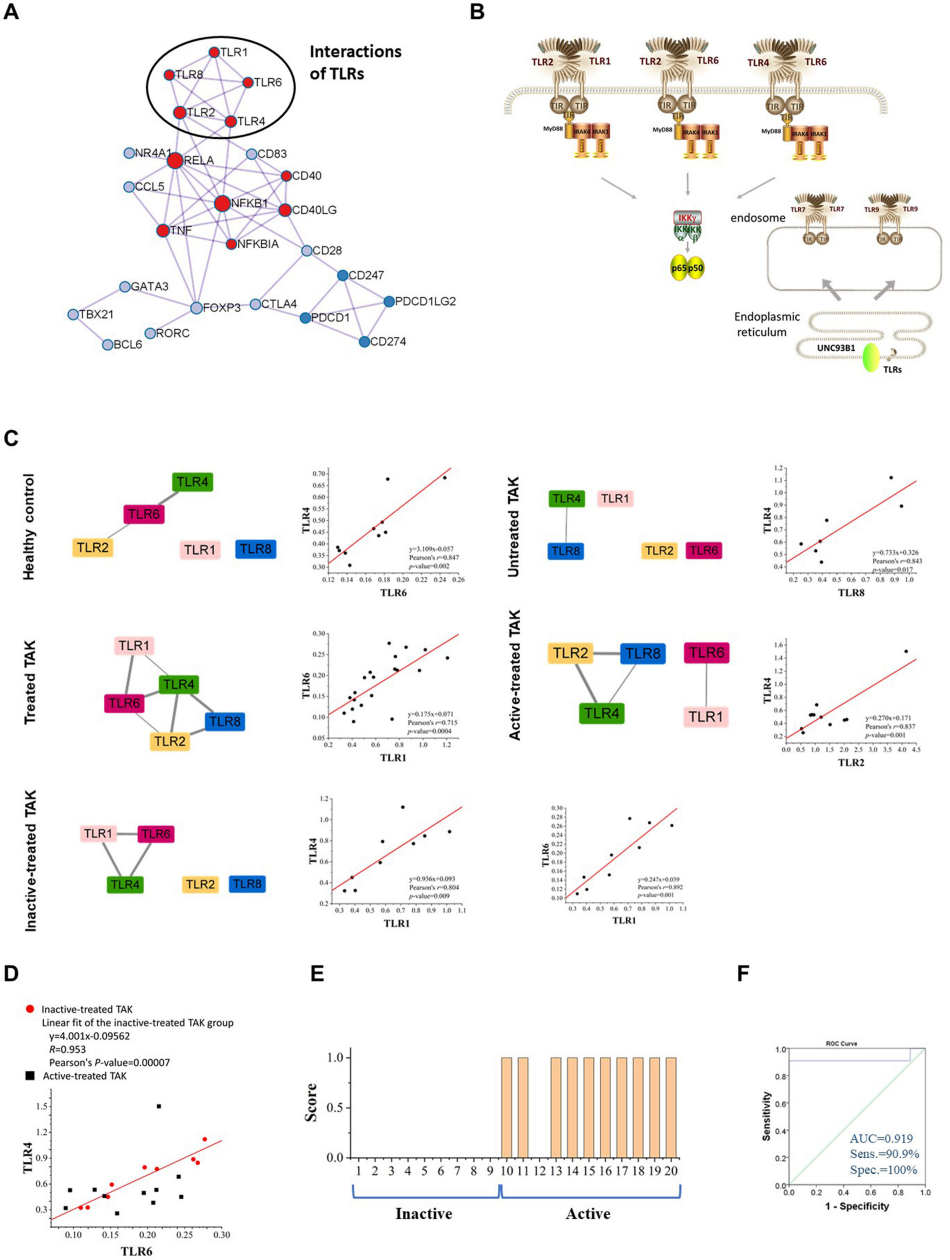
TLR-expression signature serves as a biomarker of different disease and treatment stages in TAK. **(A)** The protein-protein interaction network using the molecular complex detection (MCODE) algorithm performed by Metascape database, indicating the tight relationship among TLRs. **(B)** The schematic drawing of the interaction or cross-talk between TLRs. **(C)** The TLR-expression signature of different disease and treatment stages of TAK and scatter plots, and the different co-expressions might mean different functional relationships between TLRs at different stages. **(D, E)** The regression equation relating the TLR6 mRNA level to the TLR4 mRNA level serves as a biomarker of active disease in treated TAK. The scatter plots **(D)**, the linear regression **(D)**, the score for the disease activity assessment of each treated TAK patient **(E)**, and the receiver operating characteristic curve (ROC) **(F)** of the TLR4-TLR6 pair. TAK, Takayasu’s arteritis.

There was a tight interplay among TLRs. **Figure 6A** was the PPI network based on the STRING database analysis. TLRs function as a homodimer or heterodimer, such as TLR1/TLR2, TLR2/TLR6, and TLR4/TLR6. Besides, there is some cross-talk between TLRs, for example, TLR7 and TLR9 (**Figure 6B**). Interestingly, the co-expressed-TLR-pairs in different groups were different (**Figure 6C**). The inactive-treated TAK group had high co-expression of TLR1 and TLR4 (*r*=0.804, *p*=0.009) which was absent in the active-treated TAK group, the untreated TAK group, or the HC group. The high co-expression of TLR4 and TLR6 (*r*=0.892, *p*=0.001) exist in the HC group (*r*=0.847, *p*=0.002) as well as the inactive-treated TAK group (*r*=0.892, *p*=0.001), while was absent in the active-treated TAK group. The different co-expressions might mean different functional relationships between TLRs at different stages.

Studies have found that a heterodimer of TLR4 and TLR6 promote a protracted sterile inflammatory response after being triggered by oxidized low-density lipoprotein (LDL) and β-amyloid, which involves the pathogenesis of atherosclerosis and Alzheimer’s disease (48–50). We found that the regression equation relating the TLR6 mRNA level to the TLR4 mRNA level might be a biomarker of active disease in treated TAK. **Figure 6D** showed the scatter plots illustrating the difference in the co-expression of TLR4 and TLR6 between the inactive-treated TAK and the active-treated TAK. The points of the active-treated TAK group, except the point presented Patient No. 12, were scattered around the fitted line of the scatter points of the inactive-treated TAK group. The regression model was established and the threshold of *M*-value for assessing the disease activity was set to 0.173 according to the Youden index. Patients with an *M*-value of more than 0.173 got one score and were assessed as being in the active stage. And the AUC, the sensitivity, specificity, positive predictive value, and negative predictive value were 0.919, 100%, 90.9%, 100%, and 90%, respectively (**Figures 6E, F**). The details of the assessment were shown in **Supplementary Table 6**. The results indicated that the inactive stage of TAK could be characterized from the treated TAK by the co-expression of several TLR genes.

#### Genes Key to the Cross-Talk Between TLRs and the Activation and Differentiation of T-Cell in TAK

To identify genes closely related to TLRs in these genes key to the activation and differentiation of T-cells, we assessed the degree of functional association between the other genes were to TLRs, and the evaluation protocol is described in *Method*. Specific scores are shown in **Table 3** and the results are detailed below. In inactive-treated TAK, most of the genes exhibited functional association with TLRs. The 7 genes with the highest scores were BCL6 (12), CCL5 (11), FOXP3 (7), GATA3 (7), CD28 (6), T-bet (6), and NR4A1 (6) according to Pearson correlation analysis, and the 5 genes with the highest scores were BCL6 (16), TIGIT (14), IκBα (13), NR4A1 (12), and FOXP3 (11) according to Spearman correlation analysis. However, in the active-treated group, the functional association between these genes and TLRs did not seem to be as strong as the inactive-treated group (**Table 3**). The 3 genes with the highest scores were BCL6 (7), PD-1 (1), and LAG3 (1) according to Pearson correlation analysis, and the 5 genes with the highest scores were BCL6 (3), CD40 (3), and LAG3 (3) according to Spearman correlation analysis.

**Table 3:** Evaluation the closeness of gene-to-gene functional relationships between TLRs and the other genes.

	Pearson correlation					Spearman correlation			
Gene	The number of co-expressed TLR-genes	The number of common neighborhoods with TLRs	The number of TLRs in the co-expression cluster	Score	Gene	The number of co-expressed TLR-genes	The number of common neighborhoods with TLRs	The number of TLRs in the co-expression cluster	Score
***Treated patients with inactive TAK***								
BCL6	4	4	4	12	BCL6	4	8	4	16
CCL5	5	6	0	11	TIGIT	4	6	4	14
FOXP3	3	4	0	7	IκBα	4	5	4	13
GATA3	3	4	0	7	NR4A1	2	6	4	12
CD28	2	4	0	6	FOXP3	3	8	0	11
T-bet	3	3	0	6	CCL5	5	5	0	10
NR4A1	3	2	1	6	CD28	3	6	0	9
CD3	1	3	0	4	GATA3	3	4	0	7
CD40	0	0	4	4	T-bet	3	2	0	5
TIGIT	0	3	1	4	TCR	1	4	0	5
TCR	0	3	0	3	CD40L	0	5	0	5
CD40L	0	3	0	3	CD83	1	3	0	4
CTLA4	0	2	0	2	PD-L2	0	3	1	4
PD-1	1	0	1	2	LAG3	0	0	4	4
PD-L2	0	2	0	2	TNF	1	3	0	4
TIM3	0	1	1	2	CD3	0	2	0	2
CD83	0	1	0	1	P50	0	2	0	2
IκBα	0	1	0	1	CD40	0	1	0	1
P50	0	1	0	1	CTLA4	0	1	0	1
RORC	0	1	0	1	PD-L1	0	1	0	1
LAG3	0	0	1	1	P65	0	0	0	0
TNF	0	1	0	1	PD-1	0	0	0	0
P65	0	0	0	0	RORC	0	0	0	0
PD-L1	0	0	0	0	TIM3	0	0	0	0
***Treated patients with active TAK***								
BCL6	2	0	5	7	BCL6	0	1	2	3
PD-1	0	1	0	1	CD40	0	1	2	3
LAG3	0	1	0	1	LAG3	1	0	2	3
CD28	0	0	0	0	PD-L2	0	0	2	2
CD3	0	0	0	0	CD83	1	0	0	1
CD40	0	0	0	0	IκBα	1	0	0	1
CD83	0	0	0	0	P50	0	1	0	1
CTLA4	0	0	0	0	TIGIT	0	1	0	1
FOXP3	0	0	0	0	TIM3	0	1	0	1
GATA3	0	0	0	0	TNF	0	1	0	1
IκBα	0	0	0	0	NR4A1	0	1	0	1
P50	0	0	0	0	CD28	0	0	0	0
P65	0	0	0	0	CD3	0	0	0	0
PD-L1	0	0	0	0	CTLA4	0	0	0	0
PD-L2	0	0	0	0	FOXP3	0	0	0	0
RORC	0	0	0	0	GATA3	0	0	0	0
T-bet	0	0	0	0	P65	0	0	0	0
TCR	0	0	0	0	PD-1	0	0	0	0
TIGIT	0	0	0	0	PD-L1	0	0	0	0
TIM3	0	0	0	0	RORC	0	0	0	0
CCL5	0	0	0	0	T-bet	0	0	0	0
CD40L	0	0	0	0	TCR	0	0	0	0
TNF	0	0	0	0	CCL5	0	0	0	0
NR4A1	0	0	0	0	CD40L	0	0	0	0

BCL6, BCL6 transcription repressor; CD3, CD247; PD-1, programmed cell death 1, also known as PDCD1; PD-L1, CD274; PD-L2, PDCD1LG2; LAG3, lymphocyte activating 3; CTLA4, cytotoxic T-lymphocyte associated protein 4; FOXP3, forkhead box P3; GATA3, GATA binding protein 3; IκBα, NFκB inhibitor alpha; NFKB1 NFKB1, nuclear factor kappa B (NFκB) subunit 1, also known as p50; RELA, RELA proto-oncogene NFκB subunit, also known as p65; PD-L1, CD274; PD-L2, programmed cell death 1 ligand 2 also known as PDCD1LG2; RORC, RAR related orphan receptor C; T-bet, T-box transcription factor 21, also known as TBX21; TCR, T cell receptor; TIGIT, TNF superfamily member 14; HAVCR2, hepatitis A virus cellular receptor 2, also known as TIM3; CCL5, C-C motif chemokine ligand 5; CD40L, CD40LG; TNF, tumor necrosis factor;TAK, Takayasu’s arteritis; TLRs, Toll-like receptors.

**Figure 7:**
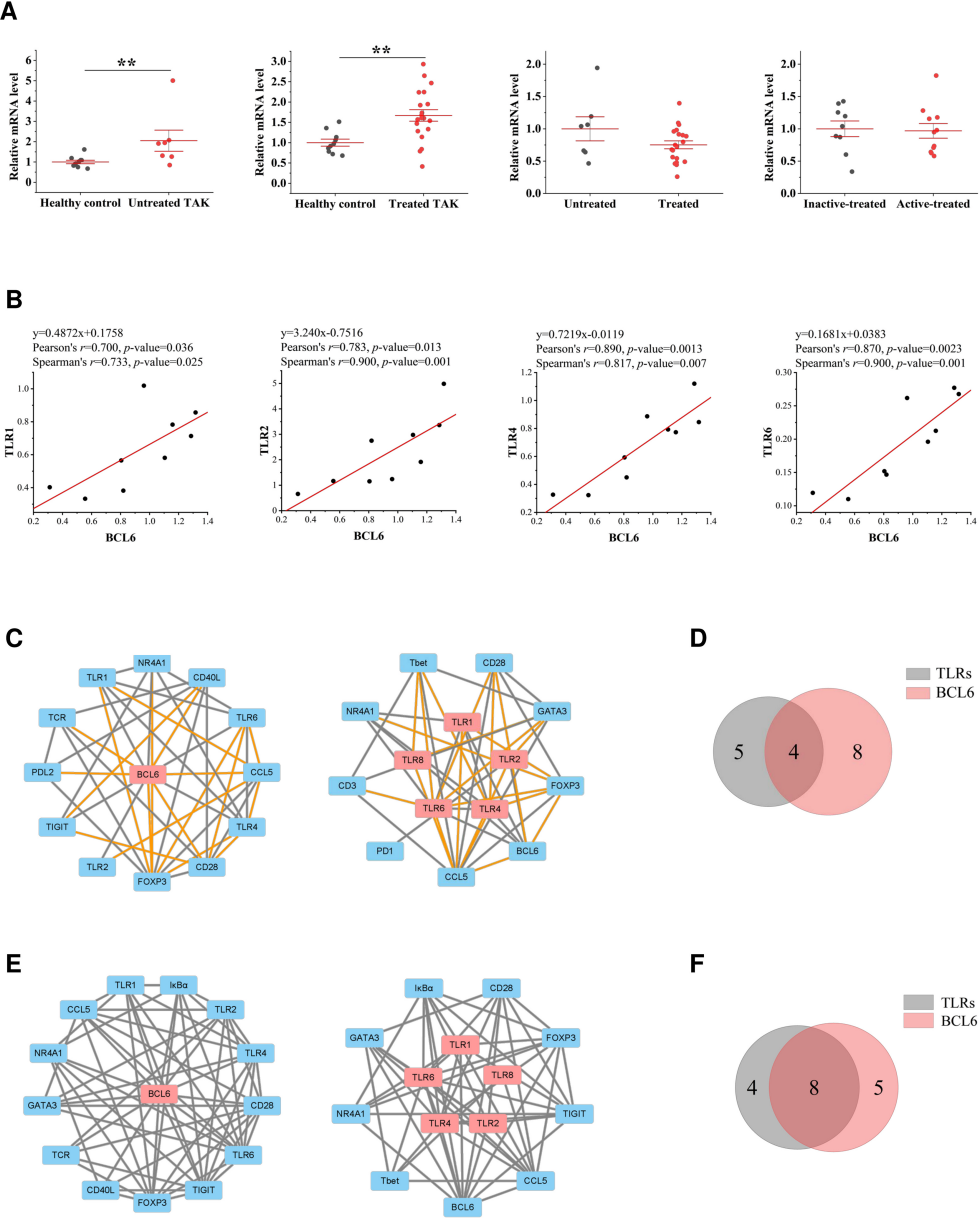
The close functional relationship between BCL6 and Toll-like receptors (TLRs) in inactive-treated TAK. **(A)** Gene expression differential analysis. Mann–Whitney test. **p < 0.01. The red center line represented the mean value of the mRNA level, and the error bar showed the standard deviation. **(B)** The scatter plots and the linear regression of the BCL6-TLR1 pair, the BCL6-TLR2 pair, the BCL6-TLR4 pair, and the BCL6-TLR6 pair in the inactive-treated TAK group, indicating that BCL6 was co-expressed with multiple TLRs. **(C)~(F)** BCL6 and TLRs shared multiple co-expressed genes. **(C)** The gene co-expression network consisting of BCL6 (left panel) or TLRs (right panel) and its/their neighborhoods based on Pearson correlation. **(D)** Intersections of the neighborhood-gene between BCL6 and TLRs based on Pearson correlation. **(E)** The gene co-expression network consisting of BCL6 (left panel) or TLRs (right panel) and its/their neighborhoods based on Spearman correlation. **(F)** Intersections of the neighborhood-gene between BCL6 and TLRs based on Spearman correlation. Healthy controls, n=10 people. Untreated TAK, n=7 people. Treated TAK, n=20 people. Active-treated TAK. n=11 people. Inactive-treated TAK, n=9 people. TAK, Takayasu’s arteritis.

Due to space restrictions, we only showed the visualized results of BCL6 and FOXP3. BCL6 is the master transcriptional regulator of Tfh cell differentiation, which is required for germinal center formation and antibody affinity maturation (51). As gene expression differential analysis showed, compared to the healthy controls, both the untreated TAK group (*p*=0.007) and the treated TAK group (*p*=0.006) had an increased mRNA level of BCL6 (**Figure 7A**). In the inactive-treated TAK group, BCL6 was co-expressed with TLR1 (Pearson’s *r*=0.700, *p*=0.036. Spearman’s *r*=0.733, *p*=0.025), TLR2 (Pearson’s *r*=0.783, *p*=0.013. Spearman’s *r*=0.900, *p*=0.001), TLR4 (Pearson’s *r*=0.890, *p*=0.0013. Spearman’s *r*=0.817, *p*=0.007), and TLR6 (Pearson’s *r*=0.870, *p*=0.0023. Spearman’s *r*=0.900, *p*=0.001) (**Figure 7B**). In the inactive-treated TAK group, according to Pearson correlation analysis, BCL6 and TLR1/2/4/6/8 shared 4 co-expressed genes, including FOXP3, CCL5, NR4A1, and CD28, and according to Spearman correlation analysis, BCL6 and TLR1/2/4/6/8 shared 8 co-expressed genes, including FOXP3, IκBα, TIGIT, CCL5, NR4A1, CD28, GATA3, and TCR (**Figures 7C–F**).

**Figure 8:**
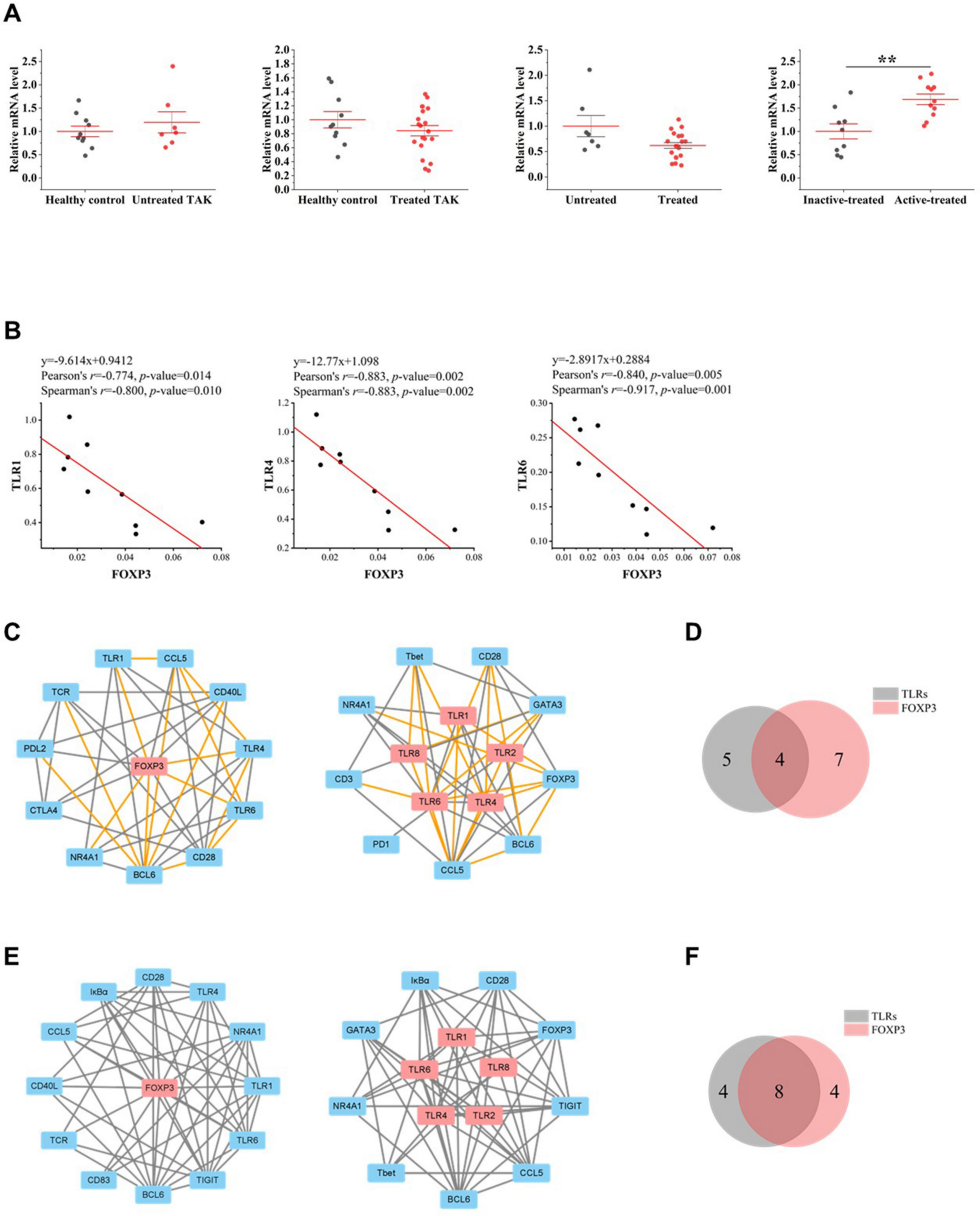
The close functional relationship between FOXP3 and Toll-like receptors (TLRs) in inactive-treated TAK. **(A)** Gene expression differential analysis. Mann–Whitney test. **p < 0.01. The red center line represented the mean value of the mRNA level, and the error bar showed the standard deviation. **(B)** The scatter plots and the linear regression of the FOXP3-TLR1 pair, the FOXP3-TLR4 pair, and the FOXP3-TLR6 pair in the inactive-treated TAK group, indicating that FOXP3 was co-expressed with multiple TLRs. **(C)~(F)** FOXP3 and TLRs shared multiple co-expressed genes. **(C)** The gene co-expression network consisting of FOXP3 (left panel) or TLRs (right panel) and its/their neighborhoods based on Pearson correlation. **(D)** Intersections of the neighborhood-gene between FOXP3 and TLRs based on Pearson correlation. **(E)** The gene co-expression network consisting of FOXP3 (left panel) or TLRs (right panel) and its/their neighborhoods based on Spearman correlation. **(F)** Intersections of the neighborhood-gene between FOXP3 and TLRs based on Spearman correlation. Healthy controls, n=10 people. Untreated TAK, n=7 people. Treated TAK, n=20 people. Active-treated TAK. n=11 people. Inactive-treated TAK, n=9 people. TAK, Takayasu’s arteritis.

FOXP3 is a transcriptional regulator which is crucial for the development and inhibitory function of regulatory T-cells (Treg) (52). As gene expression differential analysis showed, compared to the inactive-treated TAK group, the active-treated TAK group had an increased mRNA level of FOXP3 (*p*=0.004) (40) (**Figure 8A**). In the inactive-treated TAK group, FOXP3 was co-expressed with TLR1 (Pearson’s *r*=0.774, *p*=0.014. Spearman’s *r*=0.800, *p*=0.010), TLR4 (Pearson’s *r*=0.883, *p*=0.0002. Spearman’s *r*=0.883, *p*=0.002), and TLR6 (Pearson’s *r*=0.840, *p*=0.005. Spearman’s *r*=0.917, *p*=0.001) (**Figure 8B**). In the inactive-treated TAK group, according to Pearson correlation analysis, BCL6 and TLR1/2/4/6/8 shared 4 co-expressed genes, including BCL6, CCL5, NR4A1, and CD28, and according to Spearman correlation analysis, BCL6 and TLR1/2/4/6/8 shared 8 co-expressed genes, including BCL6, IκBα, TIGIT, CCL5, NR4A1, CD28, CD83, and TCR (**Figures 8C–F**).

NR4A1 is a key transcription factor that drives T cell dysfunction and plays an important role in the apoptosis of self-reactive T cells (53, 54). The results suggested that NR4A1 is likely to be functionally related to TLRs in TAK. Compared to the healthy controls, the untreated TAK group (*p*=0.0001) had an increased mRNA level of NR4A1, and that compared to the untreated TAK group, the treated TAK group (*p*=0.000005) had a decreased mRNA level of NR4A1 (**Supplementary Figure 3**).

The details of the assessment were provided in **Supplementary Table 7**.

### Different Signaling Pathways at Distinct Stages in TAK

**Figure 9:**
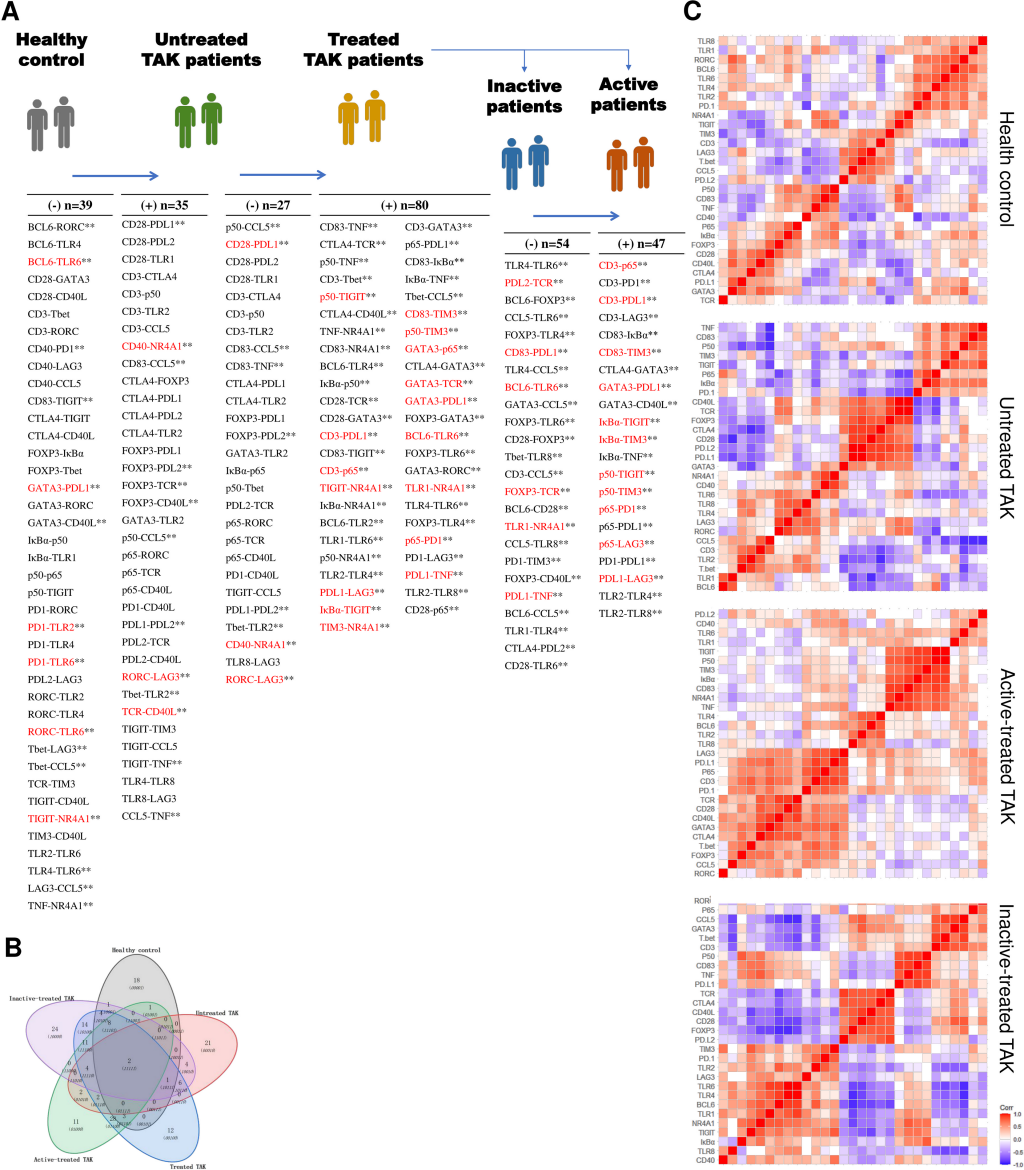
Characteristic gene co-expressions across different conditions. **(A)** A list of characteristic co-expressed gene pairs comparing the two adjacent groups, which reflected changes in the activated signaling pathways. The newly discovered co-expression relationships which had never been reported in STRING or Coexpedia database were indicated red. Pearson correlation analysis. Untreated TAK Vs. healthy controls, *p* < 0.05. Treated TAK Vs. Untreated TAK, *p* < 0.01. Active-treated TAK Vs. inactive-treated TAK, *p* < 0.01. **p < 0.01. **(B)** Overlapping co-expressions among the five conditions. **(C)** Hierarchical clustering heatmaps of the target genes with the distance calculated using Pearson correlation. TAK, Takayasu’s arteritis.

To explore the possible mechanism and signaling pathways, we summarized the co-expression variations across different conditions, which reflected changes in the activated signaling pathways (**Figure 9A**). Compared with the HC group, the untreated TAK group had 35 gene co-expression relations uniquely and lost 39 gene co-expression relations. Compared with the untreated TAK group, the treated TAK group had 80 gene co-expression relations uniquely and lost 27 gene co-expression relations. Compared with the inactive-treated TAK group, the active-treated TAK group had 47 gene co-expression relations uniquely and lost 54 gene co-expression relations. As the treated TAK group, the inactive-treated TAK group, and the active-treated TAK group had so many gene co-expression relations that the relations with a *p*-value less than 0.01 were listed only, and the complete list was shown in **Supplementary Table 8**. We also built a Venn diagram to visualize overlapping co-expressions among the five conditions (**Figure 9B**). Besides, the newly discovered co-expression relationships which had never been reported in STRING or Coexpedia database were indicated red in **Figure 9A**. The results showed the differences in signaling pathways at distinct stages.

Next, to classify the genes based on function, the heatmaps of hierarchical clustering based on the Pearson correlation were conducted (**Figure 9C**). The heatmap of the treated TAK group was shown in **Supplementary Figure 4**. Notably, the five heatmaps of five groups had different clustering structures. The two largest clusters of each group for the subsequent analyses are detailed below.

**Figure 10:**
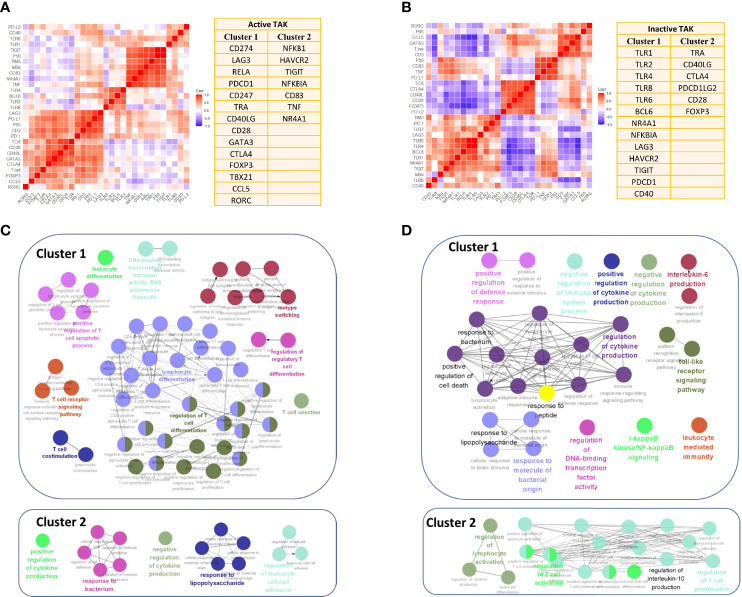
Enrichment for activated signaling pathways of the inactive-treated and the active-treated TAK patients. **(A, B)** The two greatest gene clusters of the active-treated **(A)** and the inactive-treated TAK group **(B)**. **(C, D)** Functional enrichment analysis results of the active-treated **(C)** and the inactive-treated TAK group **(D)**, revealing the potentially activated signaling pathway at the active or the inactive stage of TAK. TAK, Takayasu’s arteritis.

1. In the active-treated TAK (**Figure 10A**).
  - Cluster 1 contained 14 genes, including CD274, LAG3, RELA, PDCD1, CD247, TRA, CD40LG, CD28, GATA3, CTLA4, FOXP3, TBX21, CCL5, and RORC.
  - Cluster 2 contained 7 genes, including NFKB1, HAVCR2, TIGIT, NFKBIA, CD83, TNF, and NR4A1.
2. In the inactive-treated TAK (**Figure 10B**).
  - Cluster 1 contained 13 genes, including TLR1, TLR2, TLR4, TLR6, TLR8, BCL6, NR4A1, NFKBIA, LAG3, HAVCR2, TIGIT, PDCD1, and CD40.
  - Cluster 2 contained 6 genes, including TRA, CD40LG, CTLA4, PDCD1LG2, CD28, FOXP3.

Additionally, we performed GO enrichment analysis to further explore the signaling pathways associated with the abovementioned clusters using Metascape database, and visualized the enrichment results using ClueGO to interrogate functionally grouped gene ontology. The enrichment results are detailed below. As the results showed, in the active-treated TAK group, genes in Cluster 1 were significantly enriched in the signaling pathways related to the activation and differentiation of T-cells, while genes in Cluster 2 were significantly enriched for the regulation of cytokine production and response to bacterium and lipopolysaccharide (**Figure 10C**). In the inactive-treated TAK group, genes in Cluster 1 were enriched for the regulation of cytokine production, the response to bacterium, peptide, and lipopolysaccharide, the TLR signaling pathway, the I-kappaB kinase/NF-kappaB signaling pathway, and the regulation of defense response, while genes in Cluster 2 were enriched for the regulation of T-cell activation and the regulation of IL-10 production (**Figure 10D**).

### miRNAs Might Play an Important Role in the Cross-Talk Between TLR and T-Cell in TAK Patients

The co-expression in Cluster 1 of the inactive-treated TAK group was not presently understood, while Cluster 1 of the active-treated TAK group was led by the activation and differentiation of T-cells. We surmised that the miRNA network might take part in the expression of these genes, which could account for the co-expression of Cluster 1 of inactive-treated TAK group. We predicted the miRNA that targeted TLR1, TLR2, TLR4, TLR6, TLR8, BCL6, NR4A1, NFKBIA, LAG3, HAVCR2, TIGIT, PDCD1, and CD40 separately using the miRDB database (44, 45). Except for LAG3 among these genes, there were multiple miRNAs for each gene. We summarized the miRNA (rather than miRNA family) that might regulate two or more genes (**Supplementary Table 9**) and visualized the miRNA-gene regulatory network (**Supplementary Figure 5**), in which miRNAs belonging to the same family were merged and represented by one node.

**Figure 11:**
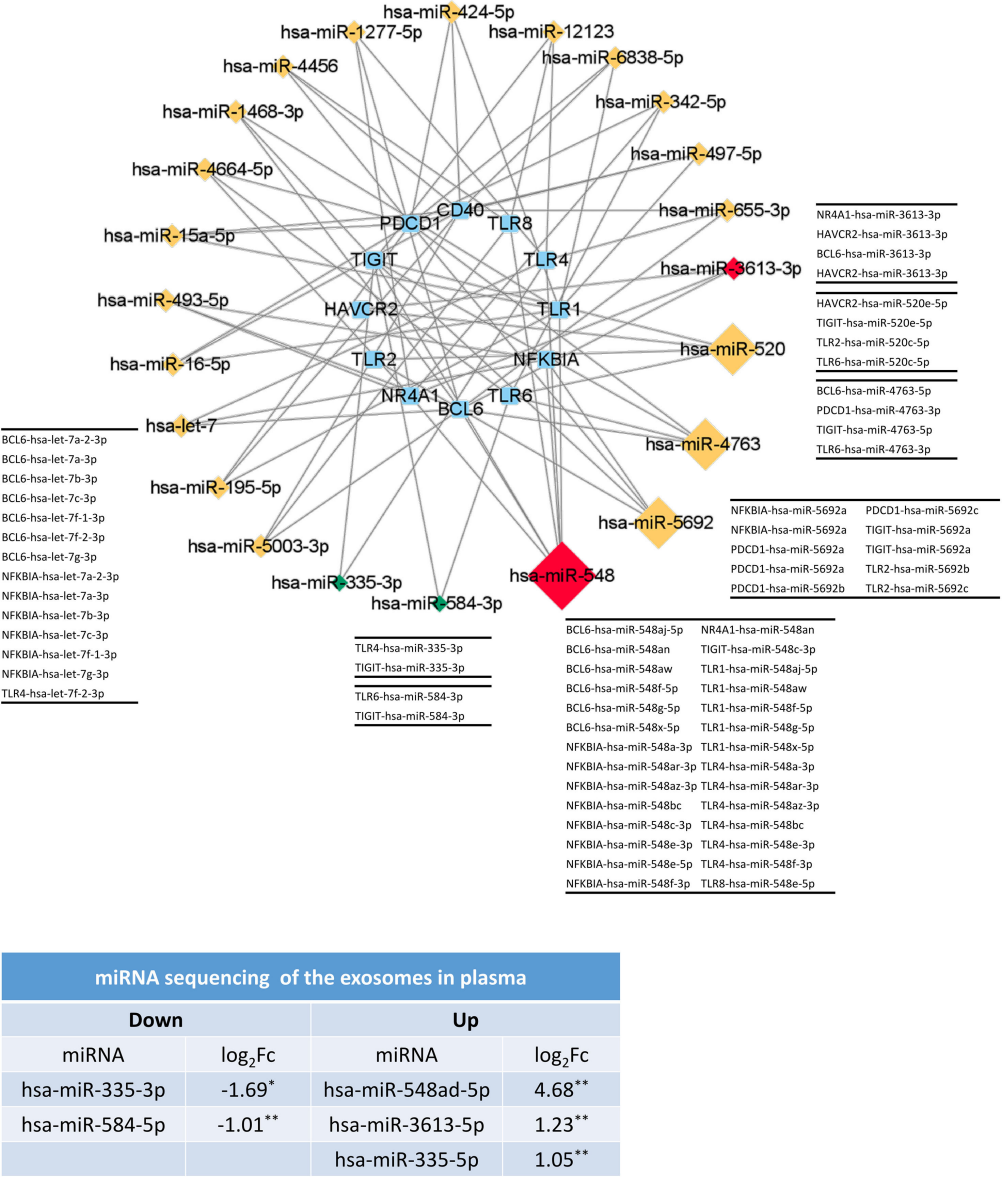
The miRNAs might play an important role in the formation of TLRs-clustering at the inactive stage of TAK. The validation results of miRNA sequencing of plasma exosomes and the predicted miRNA-gene network (miRDB database) which only included the miRNA with a degree greater than or equal to 3 or the differentially expressed miRNA. Red and green colors of miRNA indicate upregulation and downregulation, respectively. Healthy controls, n=5 people. TAK patients, n=10 people. TAK, Takayasu’s arteritis.

Next, to test whether the miRNAs in the miRNA-gene network were differentially expressed, we sequenced miRNAs from the plasma exosomes from healthy control and TAK patients. We found that compared with the healthy controls, miR-548ad-5p showed a 25.6-fold upregulation (*p*=0.0012), miR-3613-5p showed a 2.35-fold upregulation (*p*=0.0012), and miR-335-5p showed a 2.07-fold upregulation (*p*=0.0039); while miR-335-3p showed a 3.23-fold downregulation (*p*=0.042) and miR-584-5p showed a 2.01-fold downregulation (*p*=0.0092). The network shown in **Figure 11** only consisted of the miRNA that might regulate three or more genes and two differentially expressed miRNAs (miR-335-2p and miR-584-3p). In the network, miR-548 was the node with the highest degree (7 genes), followed by miR-5692 (4 genes), miR-4763 (4 genes), and miR-520 (4 genes). **Figure 11** showed the results. The miR-548 family was associated with 7 genes (7/13) in Cluster 1 of the inactive-treated TAK group, including TLR1, TLR2, TLR4, TLR6, TLR8, BCL6, NFKBIA, NR4A1, and TIGIT, which suggested that the miR-548 family plays an important role in the co-expression of Cluster 1 of inactive-treated TAK group.

**Figure 12:**
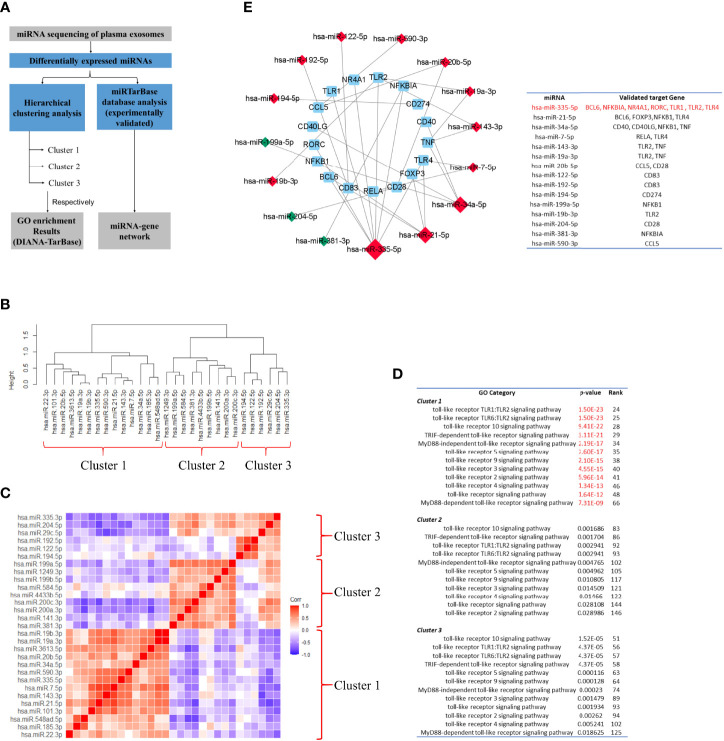
Toll-like receptor signaling pathway might be one of the major targets of the differentially-expressed-miRNA-mediated regulation. **(A)** Workflow of miRNA sequencing analysis. miRNA was from plasma exosomes in Takayasu’s arteritis (TAK) patients (n=10) and healthy controls (n=5). **(B)** The hierarchical clustering trees of miRNA expression level based on Spearman correlation coefficient using complete method. **(C)** The hierarchical clustering heatmaps of miRNA expression level based on Spearman correlation coefficient. **(D)** GO enrichment analysis for each cluster based on DIANA-TarBase, an experimentally validated database. The results indicated that TLR signaling pathway might be one of the major targets of the differentially expressed miRNA-mediated regulation, especially of the miRNAs belonging to Cluster 1. **(E)** Differentially expressed miRNAs. The miRNA-Gene-network based on miRTarBase database, an experimentally validated database, and the screening condition of “support type” was set to “Functional MTI (miRNA target-interactions). Red and green colors of miRNA indicated upregulation and downregulation, respectively. The results demonstrated that differentially expressed miRNAs that targeted multiple selected genes do exist in TAK, and TLRs, BCL6, and FOXP3 might be regulated by common miRNAs in TAK.

miRNA sequencing identified 29 differentially expressed miRNAs, 17 of which were increased and 12 were decreased. To validate whether the TLR signaling pathway might be regulated by these differentially expressed miRNAs, we performed miRNA sequencing analysis following the workflow in **Figure 12A**. First, to identify whether the differentially expressed miRNAs could be associated with specific functional categories, we performed an unsupervised hierarchical clustering of miRNA expression level based on Spearman correlation coefficient and conducted GO enrichment analysis for each cluster based on DIANA-TarBase, an experimentally validated database (55). **Figure 12B** showed miRNAs were partitioned into three clusters by cutting the clustering tree at the height of 1.1, and miRNAs belonging to the same cluster might have a close functional association. **Figure 12C** showed the hierarchical clustering heat map. The functional enrichment analysis was described as follows.

1. In Cluster 1, the most highly significant GO term was cellular nitrogen compound metabolic process (*p*-value= 9.44E-20), followed by small molecule metabolic process (*p*-value= 8.68E-15) and biosynthetic process (*p*-value= 9.03E-14). Notably, twelve ‘TLR signaling pathway’‐related GO terms were highly enriched in Cluster 1 (from 25th to 66th).
2. In Cluster 2, the most highly significant GO term was organelle (1.29E-185), followed by cellular nitrogen compound metabolic process (1.46E-92) and ion binding (1.07E-66). And twelve ‘TLR signaling pathway’‐related GO terms were highly enriched in Cluster 1 (from 83rd to 146th).
3. In Cluster 1, the most highly significant GO term was organelle (3.89E-189), followed by cellular nitrogen compound metabolic process (9.33E-102) and biosynthetic process (2.21E-67). And twelve ‘TLR signaling pathway’‐related GO terms were highly enriched in Cluster 1 (from 51st to 125th) (**Figure 12D**).

The experimentally validated database analysis indicated that TLR signaling pathway might be one of the major targets of the differentially expressed miRNA-mediated regulation, especially of the miRNAs belonging to Cluster 1.

Next, to validate whether one differentially expressed miRNA could target multiple selected genes, which might be involved in the gene co-expression of the inactive-treated TAK group, we constructed the miRNA-Gene-network based on the interactions of miRNAs and genes in the miRTarBase database, another experimentally validated database (56), and the screening condition of “support type” was set to “Functional MTI (miRNA target-interactions)”. **Figure 12E** showed the result. Within the network, we identified 7 genes (including TLR1, TLR2, TLR4, BCL6, NFKBIA, NR4A1, and RORC) that had been validated to be regulated by the same miRNA, miR-335-5p, 4 genes (including TLR4, BCL6, FOXP3, and NFKB1) by miR-21-5p, and 4 genes (including CD40, CD40LG, NFKB1, and TNF) by miR-34a-5p. The results demonstrated that as follows:

1. In TAK, differentially expressed miRNAs that targeted multiple selected genes do exist.
2. TLRs, BCL6, and FOXP3 might be regulated by common miRNAs in TAK.

To sum up, these results suggested that miRNAs might play an important role in the cross-talk between TLR and T-cell in TAK patients.

## Discussion

**Figure 13:**
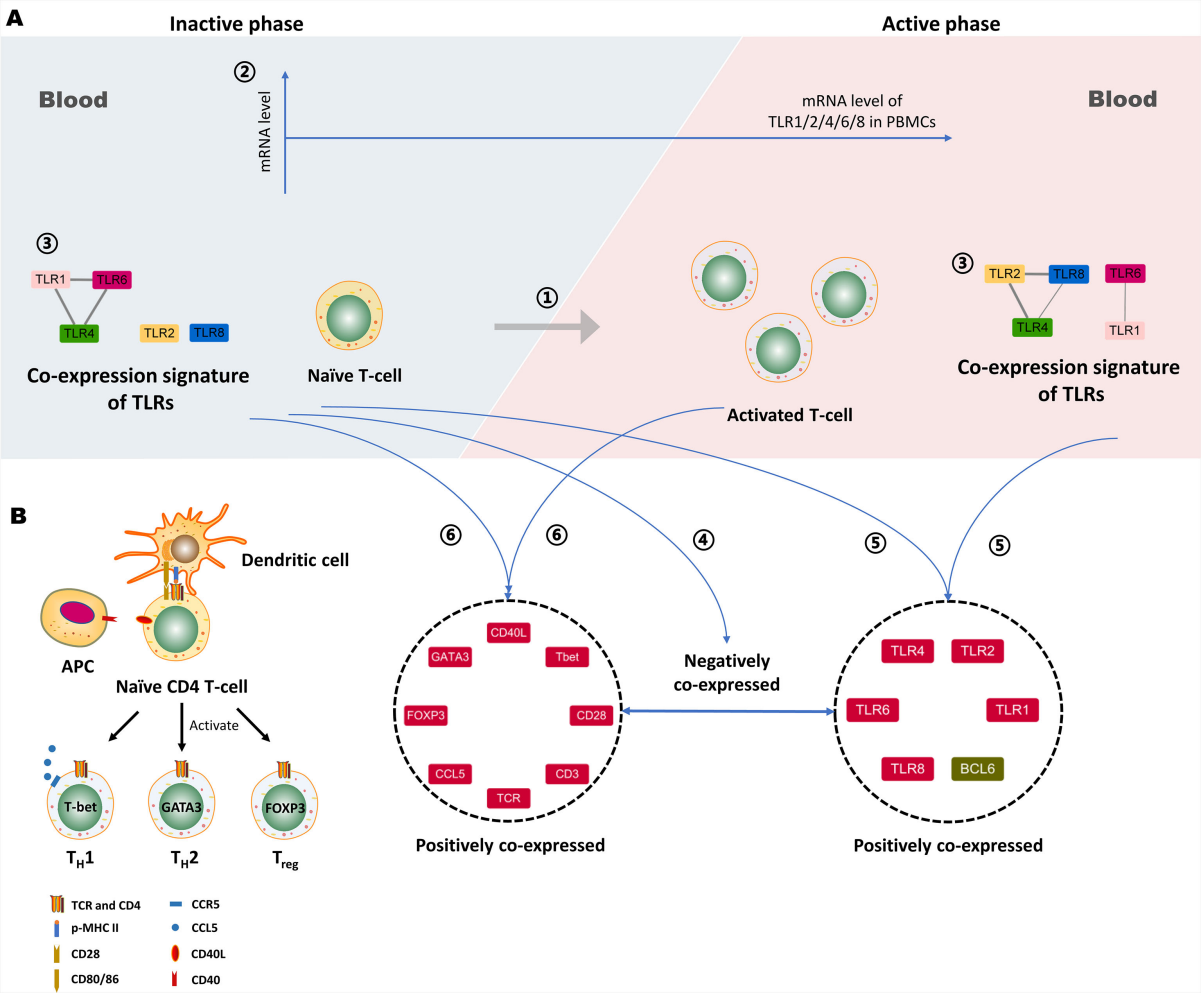
Proposed working model depicting the association between Toll-like receptors (TLRs) and T-cell activation in Takayasu’s arteritis (TAK). **(A)** ① The activation of autoreactive T cells contributes to the disease activity of TAK. ② The mRNA level of TLR1, -2, -4, -6 or, -8 in peripheral blood mononuclear cells (PBMCs) do not statistically change with alterations when patients with TAK enter the active phase, which was concluded from the comparison between the active patients and the inactive ones. ③ The gene co-expression of TLRs might serve as a biomarker that could distinguish active patients from inactive patients in TAK. ④ The negative gene co-expression between the TLRs cluster and the gene cluster associated with T-cell activation and differentiation is a characteristic of inactive-treated TAK patients that is absent in the active ones. ⑤ The inactive TAK patients and the active TAK patients share the characteristic of the positive co-expressed gene cluster comprising several key genes involved in the activation and differentiation of T-cells. ⑥ The inactive TAK patients and the active TAK patients have the common positive co-expressed gene cluster comprising TLRs and BCL6. Notably, BCL6 is tightly related to TLRs, and the underlying mechanisms deserve further exploration. Besides, miRNAs might play an important role in the above gene co-expression, and however, further investigation is needed to validate it. **(B)** The proteins coded by the genes in the left gene cluster play a key role in the activation and differentiation of T-cells. APC, Antigen-presenting cell.

There have been a number of studies demonstrating that TLRs play an important role in the pathogenesis of many AIDs, such as RA, SLE, and MS, but in TAK, it is currently unclear whether TLRs are associated with the disease activity (1, 2). T-cell is one of the major driving forces for the inflammatory response in TAK progression (6). It has been currently unknown whether TLRs are related to the disease activity or the activation and differentiation of T-cells in TAK. In this work, we selected 29 genes that were functionally enriched for the TLR signaling pathway and the activation and differentiation of T-cells. Twenty-seven TAK patients were enrolled which were grouped into the untreated and the treated group (both were further separated into the inactive and the active group), and 10 adult healthy controls were included. The relative mRNA level data of PBMCs were acquired by RT-qPCR. First, differential gene expression analysis showed that the untreated TAK and the treated TAK had an increased mRNA level of TLR2 and TLR4 compared to healthy controls. A sample-to-sample matrix revealed that 80% of healthy controls could be separated from the TAK patients. These findings suggested that TAK patients had a different expression pattern of the selected genes from the healthy controls. Second, we identified the association between TLRs and the disease activity, as the linear regression models consisting of the TLR4-CCL5 pair, the TLR6-CCL5 pair, the TLR8-CCL5 pair, and the TLR8-T-bet pair could distinguish between active and inactive disease in TAK [the AUC/sensitivity/specificity, 0.939/90.9%/88.9%]. Third, we identified the association between TLRs and the activation and differentiation of T-cells in TAK according to the following evidence: (1) As the dynamic gene co-expression network showed, compared with the healthy control group, the active-treated TAK group, and the inactive-treated TAK group had higher network connectivity, shorter characteristic path length, and a larger clustering coefficient, indicating the more active communication between TLRs and T-cell activation in TAK. (2) The inactive-treated TAK group exhibited a unique pattern of inverse correlations between the TLRs gene clusters (including TLR1/2/4/6/8, BCL6, TIGIT, NR4A1, and so on) and the gene cluster associated with T-cell activation and differentiation (including TCR, CD28, T-bet, GATA3, FOXP3, CCL5, and so on). Fourth, we explored the genes key to the cross-talk between TLRs and the activation and differentiation of T-cell in TAK. In inactive-treated TAK, BCL6, CCL5, FOXP3, GATA3, CD28, T-bet, TIGIT, IκBα, and NR4A1 were likely to have a close functional relation with TLRs. However, in the active-treated group, the association between these genes and TLRs did not seem to be as strong as the inactive-treated group, BCL6, PD-1, LAG3, and CD40 were likely to have a close functional relation with TLRs. Fifth, we analyzed the activated signaling pathways in the inactive-treated and the active-treated TAK group. Last, we predicted the miRNAs that involved the greatest cluster of the inactive-treated TAK group and validated that miRNA might play an important role in the cross-talk between TLR and T-cell in TAK by miRNA sequencing. Besides, we proposed a new concept of the TLR-co-expression signature which might distinguish different disease and treatment stages of TAK, such as the co-expression of TLR4 and TLR6, which serves as a biomarker of the active stage in treated TAK (AUC/sensitivity/specificity, 0.919/100%/90.9%). These findings were schematically summarized in **Figure 13**.

### Elevated Levels of TLR2 and TLR4 in PBMCs From TAK Patients

An increased level of TLRs in PBMCs has been detected in some AIDs. For example, comparing SLE patients with healthy controls, TLR2 expression on monocytes was reduced, and intracellular TLR9 expression of CD19^+^ B-cells was elevated (57). Comparing Behcet’s disease patients with healthy controls, TLR1 and TLR2 were elevated in B-cells, TLR1, 2, and 4 were more highly expressed in both CD4^+^and CD8^+^ T-cells, granulocytes displayed a higher expression of TLR1, 2, 4, and 6, and the expressions of TLR2, 4, and 5 were significantly increased on classical monocytes (58). However, few studies probed the effect of the treatment on the expressions of the TLRs in AIDs. For TAK, it has been unclear whether there are abnormal expressions of the TLRs in TAK, except TLR4. Besides, it also has been unclear whether the abnormal expressions of the TLRs are related to the medication and the disease activity, including TLR4. Our results complement and add to findings from previous TAK studies in that the mRNA expression of TLR4 and its ligand S100s in PBMCs from TAK patients were higher compared to healthy controls (12). We found that both the untreated and the treated TAK patients had increased mRNA levels of TLR2 and TLR4, and there was no statistically significant difference between the two groups, which demonstrated that the increased mRNA levels of TLR2 and TLR4 were independent from the medication. Besides, it was unanticipated that there was no statistically significant difference between the inactive and the active TAK patients in the mRNA expression of TLR2 and TLR4, which suggested that the elevated levels of TLR2 and TLR4 might be not resulted from the disease activity in TAK. However, the treated patients in this study had a relatively low serum level of TNF-α, which might imply relatively inactive TLR-signaling pathways in their peripheral blood.

### Novel Association Between TLRs and Disease Activity in AIDs

Many studies have shown that TLRs play a dual role in the disease activity of AIDs. On the one hand, TLRs promote disease progression. TLRs can be activated by the DAMPs which are released from the injured tissue, such as high mobility group box-1 (HGMB1) (59) and self-nucleic acids (60, 61). On the other hand, TLRs although inhibit autoimmune inflammation. In SLE, TLRs can promote sterile inflammation activated by DAMPs released from damaged cells, which is closely related to the disease progression and autoantibody production (62). For example, TLR-7, -8, and -9, which are not only expressed in the innate immune cells, such as neutrophils, macrophages and dendritic cells (DCs), but also are expressed in the lymphocytes, such as T-cells, and B-cells (60, 63, 64), can be activated by the self-nucleic acids (DAMPs released from damaged cells) (60, 61), and in recent years, there has been an increasing interest in the mechanism of how TLRs directly regulate the adaptive immune response without the innate immune cells, which deepens our understanding of the role of TLRs in the pathogenesis of AIDs. On the other hand, TLRs have a dual role in the pathogenesis of SLE, and TLRs can protect the body against autoimmune inflammation. Low expression of TLR9 due to single nucleotide polymorphisms would increase SLE susceptibility in humans (65), and deletion of TLR8 and TLR9 would accelerate lupus development in mice (66–70). A variety of mechanisms for TLR9 to promote and limit AIDs have been discovered. The TLR9-dependent function of medullary thymic epithelial cells is required for the generation of regulatory T cells (Tregs) and the establishment of central tolerance (71). TLR9 can inhibit the production of autoantibodies mediated by TLR7, such as anti-RNA Ab, anti-Sm Ab, anti-RNA Ab, anti-IgG2a RF (68, 69, 72). The balance between TLR7 and TLR9 is important for the formation of autoreactive B cells (73). And UNC93B1 plays an important role in regulating the balance between TLR7 and TLR9 (74–77). In the endoplasmic reticulum, TLR7 competes with TLR9 to bind to UNC93B1, and the D34 position of UNC93B makes it biased to bind to TLR9 (78). In RA, TLRs also promote sterile inflammation. For example, TLR4 can be activated by high mobility group box-1 protein (HMGB1) in RA (59). Besides, the activation of TLRs caused by PAMPs also plays an important role in the pathogenesis of RA (79). In the clinic, some cytokines induced by TLRs are immunotherapeutic targets in AIDs, such as TNF-α, interleukin (IL)-6, interferon (IFN)-α, and IL-1β (7). Recently, NI-0101, an anti-toll-like receptor 4 monoclonal antibody was used in RA, which was the first clinical trial to target TLRs to treat autoimmune diseases (4). In this study, we observed a unique pattern of inverse correlations between the TLRs gene clusters and the gene cluster associated with T-cell activation and differentiation the inactive-treated TAK group, which suggests a novel relationship between TLRs and the disease activity in AIDs.

However, these potential mechanisms of these inverse correlations need to be elucidated in depth, and there are several fundamental questions to address. First, the inverse correlations between TLRs and the activation of T-cell should be described in more detailed, and some experimental data for the difference in the T-cell activation status are needed to be collected, including the expression of activation markers on T cells (such as CD69, CD25 and HLA-DR) and some cytokines (such as IFN-γ^+^Th1, IL-4^+^Th2, and IL-17^+^Th17). Then, association studies cannot be used to infer causality. While there is a possible regulatory relationship between TLRs and T-cells in TAK, it is also possible that TLRs and T-cells are under the control of common upstream regulators. Furthermore, another question is why the inverse correlations exist in inactive TAK patients, but not in active TAK patients.

### Activated Signaling Pathways in the Inactive and the Active TAK Patients

T-cell is one of the main forces for the autoimmune inflammation in TAK (6). It has been reported that multiple T-cell subtypes are related to the pathogenesis of TAK, including Th1, Th17, Th9, Tfh, and Th2-like Treg cells (80–83). In this study, the functional enrichment of the greatest cluster of the active-treated TAK group was centered on the activation and differentiation of T-cells, indicating the activation of T-cells at the active stage, which is consistent with previously reported studies. Notably, the functional enrichment of the greatest cluster of the inactive-treated TAK group was centered on the regulation of cytokine production, the response to bacterium, peptide, and lipopolysaccharide, the TLR signaling pathway, the I-kappaB kinase/NF-kappaB signaling pathway, the regulation of defense response, and so on, which suggested the activated TLR-signaling pathways at the inactive stage.

The previous study has shown that BCL6 regulates nearly a third of the TLR4-regulated transcriptome, and that 90% of the BCL6 cistrome is collapsed following TLR4 activation in the macrophages (84). In B-cells, PELI1, which is activated by TLR3 and TLR4, directly interacted with BCL6, inducing lysine 63-mediated BCL6 polyubiquitination, and PELI1 expression levels positively correlated with BCL6 expression (85). Our results showed that BCL6 had a very similar expression pattern to that of TLRs, indicating that there is a stronger and closer association between multiple TLRs and BCL6, such as direct interactions and regulatory relationships, and this association warrants further study. The transcription factor BCL6 is required for driving Tfh cell differentiation and regulates their function. Recent studies have demonstrated the implications of Tfh cells with the disease activity of TAK (82), and our results suggested that TLRs might play a role in the regulation of Tfh cells.

### miRNA-548 Family

miRNAs play an important role in regulating gene expression and the TLR-signaling pathways (86, 87). miRNAs with high sequence similarity form the miRNA family, co-regulating complex biological processes. Among them, the miR-548 family, with over 80 identified miRNAs, regulates the immune process in some diseases, such as cancer (88, 89). A large amount studies have shown that miRNA-548 suppresses tumor proliferate by binding WNT2 (90), murine double minute 2 (91), metastasis tumor-associated protein-2 (92), specificity protein 1 (93), cancerous inhibitor of protein phosphatase 2A (94), HMGB1 (95), and so on. Inhibiting or down-regulating miR-548 promotes the tumor proliferation (96). One study proposed to serve miRNA-548 as a prognosis predictor in primary central nervous system lymphoma (97). Our study showed that miR-548 played an important role in the pathogenesis of autoimmune disease, which might be related to the TLR signaling pathway.

It is worth noting that miRNA-548 family was increased in the serum exosomes from TAK patients than healthy controls, while the expression levels of its potential target genes were not decreased. While we have no clear explanation for this, we speculated that miRNAs might play an important role in the negative feedback regulation of TLR signaling pathways based on previous studies. Studies have shown that miRNAs play an important role as negative feedback to control inflammatory responses maintaining a level, avoiding excess inflammatory responses (98). For example, serum IL-6 levels increase with aging, whereas miR-192 in extracellular vesicles alleviated the prolonged inflammation associated with aging (99). Besides, the expression level of miRNA-541 [can attenuate pro-inflammatory cytokine expression (100)] in circulating extracellular vesicles was negatively correlated with the pro-inflammatory cytokine expression levels and the number of adverse local symptoms after vaccination (101). In these cases, although the expression levels of some targeted genes are still increased than normal, miRNAs in circulating extracellular vesicles have functioned as avoiding excessive inflammation. However, we have not been demonstrated the correlations between miR-548 as the RNA samples and serum exosomes were not from the same participants.

### TLR-Co-Expression Signature

There are certain functional relationships among TLRs. First, TLRs function as a homodimer or heterodimer. TLR2 binds TLR1 or TLR6 to recognize distinct PAMPs (102, 103). The TLR4-TLR6-CD36 complex is activated by atherogenic lipids and amyloid-beta to stimulate sterile inflammation (48, 49). Second, there is some cross-talk between TLRs. In SLE, TLR9 can inhibit the production of autoantibodies mediated by TLR7, such as anti-RNA Ab, anti-Sm Ab, anti-RNA Ab, anti-IgG2a RF (68, 69, 72). The balance between TLR7 and TLR9 is important for the formation of autoreactive B cells (73). And UNC93B1 plays an important role in regulating the balance between TLR7 and TLR9 (74–77). In the endoplasmic reticulum, TLR7 competes with TLR9 to bind to UNC93B1, and the D34 position of UNC93B makes it biased to bind to TLR9 (78). In the endothelial cells transfected with TLR1in septic, the ability of TLR4 in these cells to respond to LPS was lost (104). TLR10 on peripheral blood monocytes reduces TLR2-induced cytokine production in Parkinson’s disease (105), which is also a co-receptor of TLR2. Third, some TLR-genes locate the same gene cluster in the chromosome, and there are multiple associated SNPs of TLRs, such as TLR1, TLR6, and TLR10. For example, the observed multiple associated SNPs at the TLR6-TLR1-TLR10 gene cluster may play a role in prostate cancer risk (106). STRING database analysis showed strong PPIs among TLRs (**Figure 6A**). We observed the different co-expressed TLRs clusters of different disease and treatment stages in TAK, which might serve as a signature of transcriptome analysis for individualized treatment decision. For instance, the co-expression TLR4 and TLR6 could distinguish the active-treated TAK patients from the inactive ones.

### Limitations

In the correlation analysis results, there were many non-strong correlations (0.01<p<0.05) between the expression levels, which might be related to the sample heterogeneity. For instance, some non-strong correlation in the treated TAK group became strong correlations (p<or=0.01) after further stratifying patients into subgroups according to disease activity. But the sample size is relatively small, so the active or inactive group could not be separated into subgroups.

Besides, there were limitations in the protocol for evaluating the closeness of functional relationships between TLRs and the other genes. Although the genes with a high score were likely to be closely connected in function with TLRs, the genes with a low score were not necessarily intimately linked with TLRs.

Notably, the reference genes had a large influence on the correlation analysis results. Stably expressed reference genes help find out the co-expression relations. Moreover, different reference genes may cause some differences in the results, and combining Pearson correlation and Spearman correlation help partially address the problem.

Last, larger clinical studies will be needed to validate our findings and to calibrate the diagnostic thresholds. In this cohort, the naïve TAK patients will be also classified into the active group and the inactive group as it might exhibit a different co-expression network from the TAK patients under treatment. Some experimental data for the difference in the T cell activation status (activation markers and cytokines) will be also collected to construct more accurate and more detailed models of the association between TLRs and T-cell activation in TAK.

## Conclusions

This study identified the association between TLRs and T-cell activation in TAK, found a potentially novel role of TLRs in the pathogenesis of autoimmune diseases, and highlighted the function of miRNAs in the cross-talk between TLRs and T-cells in TAK, and more investigation is needed to further confirm the role of miRNAs in the cross-talk between TLR and T-cell in TAK patients and to elucidate the mechanisms.

## Data Availability Statement

We have deposited the miRNA data in the OMIX, China National Center for Bioinformation/Beijing Institute of Genomics, Chinese Academy of Sciences (https://ngdc.cncb.ac.cn/omix: accession no. OMIX807).

## Ethics Statement

The studies involving human participants were reviewed and approved by the Institutional Review Board of Peking Union Medical College Hospital. The patients/participants provided their written informed consent to participate in this study.

## Author Contributions

YT performed the RT-qPCR experiments and analyzed the data. BH performed the experiment of miRNA sequencing and differential miRNA expression analysis. JL, YT, and XT designed the study. JL and YT wrote the paper mainly. XT and XZ made the critical revision. All authors have read and approved the paper.

## Funding

This study was supported by National Science Foundation of China to JL with (grant numbers 81401333), and XZ with National Clinical Research Center for Dermatologic and Immunologic Diseases, the Chinese National Key Research and Development Program (grant number 2017YFC0907600, 2008BAI59B02) and the Chinese National High Technology Research and Development Program, Ministry of Science and Technology (grant number 2012AA02A513).

## Conflict of Interest

The authors declare that the research was conducted in the absence of any commercial or financial relationships that could be construed as a potential conflict of interest.

## Publisher’s Note

All claims expressed in this article are solely those of the authors and do not necessarily represent those of their affiliated organizations, or those of the publisher, the editors and the reviewers. Any product that may be evaluated in this article, or claim that may be made by its manufacturer, is not guaranteed or endorsed by the publisher.

## References

[1] Chen JQ Szodoray P Zeher M. Toll-Like Receptor Pathways in Autoimmune Diseases. Clin Rev Allergy Immunol (2016) 50:1–17. doi: 10.1007/s12016-015-8473-z 25687121

[2] Joosten LA Abdollahi-Roodsaz S Dinarello CA O’Neill L Netea MG. Toll-Like Receptors and Chronic Inflammation in Rheumatic Diseases: New Developments. Nat Rev Rheumatol (2016) 12:344–57. doi: 10.1038/nrrheum.2016.61 27170508

[3] Deng J Ma-Krupa W Gewirtz AT Younge BR Goronzy JJ Weyand CM. Toll-Like Receptors 4 and 5 Induce Distinct Types of Vasculitis. Circ Res (2009) 104:488–95. doi: 10.1161/circresaha.108.185777 PMC2731717 19150884

[4] Monnet E Choy EH McInnes I Kobakhidze T de Graaf K Jacqmin P. Efficacy and Safety of NI-0101, an Anti-Toll-Like Receptor 4 Monoclonal Antibody, in Patients With Rheumatoid Arthritis After Inadequate Response to Methotrexate: A Phase II Study. Ann Rheum Dis (2020) 79:316–23. doi: 10.1136/annrheumdis-2019-216487 31892533

[5] Hellmann DB. 88 - Giant Cell Arteritis, Polymyalgia Rheumatica, and Takayasu’s Arteritis. In: Firestein GS Budd RC Gabriel SE McInnes IB O’Dell JR, editors. Kelley’s of, (Ninth Edition). Philadelphia: TextbookRheumatologyW.B. Saunders (2013). p. 1461–80.

[6] Watanabe R Berry GJ Liang DH Goronzy JJ Weyand CM. Cellular Signaling Pathways in Medium and Large Vessel Vasculitis. Front Immunol (2020) 11:587089. doi: 10.3389/fimmu.2020.587089 33072134 PMC7544845

[7] Mansson A Adner M Cardell LO. Toll-Like Receptors in Cellular Subsets of Human Tonsil T Cells: Altered Expression During Recurrent Tonsillitis. Respir Res (2006) 7:36. doi: 10.1186/1465-9921-7-36 16504163 PMC1481585

[8] Ye J Wang Y Liu X Li L Opejin A Hsueh EC. TLR7 Signaling Regulates Th17 Cells and Autoimmunity: Novel Potential for Autoimmune Therapy. J Immunol (Baltimore Md 1950) (2017) 199:941–54. doi: 10.4049/jimmunol.1601890 PMC5542577 28652396

[9] Marks KE Flaherty S Patterson KM Stratton M Martinez GJ Reynolds JM. Toll-Like Receptor 2 Induces Pathogenicity in Th17 Cells and Reveals a Role for IPCEF in Regulating Th17 Cell Migration. Cell Rep (2021) 35:109303. doi: 10.1016/j.celrep.2021.109303 34192530 PMC8270556

[10] Li L Liu X Sanders KL Edwards JL Ye J Si F. TLR8-Mediated Metabolic Control of Human Treg Function: A Mechanistic Target for Cancer Immunotherapy. Cell Metab (2019) 29:103–23.e5. doi: 10.1016/j.cmet.2018.09.020 30344014 PMC7050437

[11] Chodaczek G Pagni PP Christoffersson G Ratliff SS Toporkiewicz M Wegrzyn AS. The Effect of Toll-Like Receptor Stimulation on the Motility of Regulatory T Cells. J Autoimmun (2021) 116:102563. doi: 10.1016/j.jaut.2020.102563 33189487

[12] Kabeerdoss J Thomas M Goel R Mohan H Danda S Jeyaseelan L. High Expression of S100 Calgranulin Genes in Peripheral Blood Mononuclear Cells From Patients With Takayasu Arteritis. Cytokine (2019) 114:61–6. doi: 10.1016/j.cyto.2018.11.033 30594066

[13] Kabeerdoss J Goel R Mohan H Danda D. High Expression of Pro-Inflammatory Cytokine Genes IL-1β and IL-1R2 Upon TLR4 Activation in Takayasu Arteritis. Rheumatol Int (2021). doi: 10.1007/s00296-020-04785-0 33528653

[14] Barabási AL Oltvai ZN. Network Biology: Understanding the Cell’s Functional Organization. Nat Rev Genet (2004) 5:101–13. doi: 10.1038/nrg1272 14735121

[15] Boccaletti S Latora V Moreno Y Chavez M Hwang DU. Complex Networks: Structure and Dynamics. Phys Rep-Rev Section Phys Lett (2006) 424:175–308. doi: 10.1016/j.physrep.2005.10.009

[16] Vidal M Cusick ME Barabási AL. Interactome Networks and Human Disease. Cell (2011) 144:986–98. doi: 10.1016/j.cell.2011.02.016 PMC3102045 21414488

[17] Bray D. Molecular Networks: The Top-Down View. Sci (New York NY) (2003) 301:1864–5. doi: 10.1126/science.1089118 14512614

[18] Zhou X Kao MC Wong WH. Transitive Functional Annotation by Shortest-Path Analysis of Gene Expression Data. Proc Natl Acad Sci USA (2002) 99:12783–8. doi: 10.1073/pnas.192159399 PMC130537 12196633

[19] Butte AJ Tamayo P Slonim D Golub TR Kohane IS. Discovering Functional Relationships Between RNA Expression and Chemotherapeutic Susceptibility Using Relevance Networks. Proc Natl Acad Sci USA (2000) 97:12182–6. doi: 10.1073/pnas.220392197 PMC17315 11027309

[20] Lee HK Hsu AK Sajdak J Qin J Pavlidis P. Coexpression Analysis of Human Genes Across Many Microarray Data Sets. Genome Res (2004) 14:1085–94. doi: 10.1101/gr.1910904 PMC419787 15173114

[21] Eisen MB Spellman PT Brown PO Botstein D. Cluster Analysis and Display of Genome-Wide Expression Patterns. Proc Natl Acad Sci USA (1998) 95:14863–8. doi: 10.1073/pnas.95.25.14863 PMC24541 9843981

[22] Prieto C Risueño A Fontanillo C De las Rivas J. Human Gene Coexpression Landscape: Confident Network Derived From Tissue Transcriptomic Profiles. PloS One (2008) 3:e3911. doi: 10.1371/journal.pone.0003911 19081792 PMC2597745

[23] Wei SC Duffy CR Allison JP. Fundamental Mechanisms of Immune Checkpoint Blockade Therapy. Cancer Discov (2018) 8:1069–86. doi: 10.1158/2159-8290.Cd-18-0367 30115704

[24] Bateman A Martin MJ O’Donovan C Magrane M Apweiler R Alpi E. UniProt: A Hub for Protein Information. Nucleic Acids Res (2015) 43:D204–12. doi: 10.1093/nar/gku989 PMC4384041 25348405

[25] Zhou Y Zhou B Pache L Chang M Khodabakhshi AH Tanaseichuk O. Metascape Provides a Biologist-Oriented Resource for the Analysis of Systems-Level Datasets. Nat Commun (2019) 10:1523. doi: 10.1038/s41467-019-09234-6 30944313 PMC6447622

[26] Ashburner M Ball CA Blake JA Botstein D Butler H Cherry JM. Gene Ontology: Tool for the Unification of Biology. Nat Genet (2000) 25:25–9. doi: 10.1038/75556 PMC3037419 10802651

[27] Kanehisa M Goto S. KEGG: Kyoto Encyclopedia of Genes and Genomes. Nucleic Acids Res (2000) 28:27–30. doi: 10.1093/nar/28.1.27 10592173 PMC102409

[28] Slenter DN Kutmon M Hanspers K Riutta A Windsor J Nunes N. WikiPathways: A Multifaceted Pathway Database Bridging Metabolomics to Other Omics Research. Nucleic Acids Res (2018) 46:D661–7. doi: 10.1093/nar/gkx1064 PMC5753270 29136241

[29] Yang S Kim CY Hwang S Kim E Kim H Shim H. COEXPEDIA: Exploring Biomedical Hypotheses *via* Co-Expressions Associated With Medical Subject Headings (MeSH). Nucleic Acids Res (2017) 45:D389–d396. doi: 10.1093/nar/gkw868 27679477 PMC5210615

[30] Szklarczyk D Gable AL Lyon D Junge A Wyder S Huerta-Cepas J. STRING V11: Protein-Protein Association Networks With Increased Coverage, Supporting Functional Discovery in Genome-Wide Experimental Datasets. Nucleic Acids Res (2019) 47:D607–d613. doi: 10.1093/nar/gky1131 30476243 PMC6323986

[31] Bloch DA Michel BA Hunder GG McShane DJ Arend WP Calabrese LH. The American College of Rheumatology 1990 Criteria for the Classification of Vasculitis. Patients and Methods. Arthritis Rheum (1990) 33:1068–73. doi: 10.1002/art.1780330803 2202306

[32] Kerr G. Takayasu’s Arteritis. Curr Opin Rheumatol (1994) 6:32–8. doi: 10.1097/00002281-199401000-00006 7913334

[33] Rio DC Ares M Jr Hannon GJ Nilsen TW. Purification of RNA Using TRIzol (TRI Reagent). Cold Spring Harbor Protoc (2010) 2010:pdb.prot5439. doi: 10.1101/pdb.prot5439 20516177

[34] Vandesompele J De Preter K Pattyn F Poppe B Van Roy N De Paepe A. Accurate Normalization of Real-Time Quantitative RT-PCR Data by Geometric Averaging of Multiple Internal Control Genes. Genome Biol (2002) 3:Research0034. doi: 10.1186/gb-2002-3-7-research0034 12184808 PMC126239

[35] Andersen CL Jensen JL Orntoft TF. Normalization of Real-Time Quantitative Reverse Transcription-PCR Data: A Model-Based Variance Estimation Approach to Identify Genes Suited for Normalization, Applied to Bladder and Colon Cancer Data Sets. Cancer Res (2004) 64:5245–50. doi: 10.1158/0008-5472.Can-04-0496 15289330

[36] Pfaffl MW Tichopad A Prgomet C Neuvians TP. Determination of Stable Housekeeping Genes, Differentially Regulated Target Genes and Sample Integrity: BestKeeper - Excel-Based Tool Using Pair-Wise Correlations. Biotechnol Lett (2004) 26:509–15. doi: 10.1023/b:Bile.0000019559.84305.47 15127793

[37] Obayashi T Kinoshita K. Rank of Correlation Coefficient as a Comparable Measure for Biological Significance of Gene Coexpression. DNA Res an Int J Rapid Publ Rep Genes Genomes (2009) 16:249–60. doi: 10.1093/dnares/dsp016 PMC2762411 19767600

[38] Santos SD Takahashi DY Nakata A Fujita A. A Comparative Study of Statistical Methods Used to Identify Dependencies Between Gene Expression Signals. Briefings Bioinf (2014) 15:906–18. doi: 10.1093/bib/bbt051 23962479

[39] Kumari S Nie J Chen HS Ma H Stewart R Li X. Evaluation of Gene Association Methods for Coexpression Network Construction and Biological Knowledge Discovery. PloS One (2012) 7:e50411. doi: 10.1371/journal.pone.0050411 23226279 PMC3511551

[40] Tian Y Li J Tian X Zeng X. Using the Co-Expression Network of T Cell-Activation-Related Genes to Assess the Disease Activity in Takayasu’s Arteritis Patients. Arthritis Res Ther (2021) 23:303. doi: 10.1186/s13075-021-02636-2 34915894 PMC8675511

[41] D’Haeseleer P Liang S Somogyi R. Genetic Network Inference: From Co-Expression Clustering to Reverse Engineering. Bioinf (Oxf Engl) (2000) 16:707–26. doi: 10.1093/bioinformatics/16.8.707 11099257

[42] Proctor RH Brown DW Plattner RD Desjardins AE. Co-Expression of 15 Contiguous Genes Delineates a Fumonisin Biosynthetic Gene Cluster in Gibberella Moniliformis. Fungal Genet Biol FG B (2003) 38:237–49. doi: 10.1016/s1087-1845(02)00525-x 12620260

[43] Kalhorzadeh P Hu Z Cools T Amiard S Willing EM De Winne N. Arabidopsis Thaliana RNase H2 Deficiency Counteracts the Needs for the WEE1 Checkpoint Kinase But Triggers Genome Instability. Plant Cell (2014) 26:3680–92. doi: 10.1105/tpc.114.128108 PMC4213155 25217508

[44] Wong N Wang XW. miRDB: An Online Resource for microRNA Target Prediction and Functional Annotations. Nucleic Acids Res (2015) 43:D146–52. doi: 10.1093/nar/gku1104 PMC4383922 25378301

[45] Chen Y Wang X. miRDB: An Online Database for Prediction of Functional microRNA Targets. Nucleic Acids Res (2020) 48:D127–d131. doi: 10.1093/nar/gkz757 31504780 PMC6943051

[46] Zeng Z Lan T Wei Y Wei X. CCL5/CCR5 Axis in Human Diseases and Related Treatments. Genes Dis (2021) 9:12–27. doi: 10.1016/j.gendis.2021.08.004 34514075 PMC8423937

[47] Langfelder P Horvath S. WGCNA: An R Package for Weighted Correlation Network Analysis. BMC Bioinf (2008) 9:559. doi: 10.1186/1471-2105-9-559 PMC2631488 19114008

[48] Stewart CR Stuart LM Wilkinson K van Gils JM Deng J Halle A. CD36 Ligands Promote Sterile Inflammation Through Assembly of a Toll-Like Receptor 4 and 6 Heterodimer. Nat Immunol (2010) 11:155–61. doi: 10.1038/ni.1836 PMC2809046 20037584

[49] Shmuel-Galia L Klug Y Porat Z Charni M Zarmi B Shai Y. Intramembrane Attenuation of the TLR4-TLR6 Dimer Impairs Receptor Assembly and Reduces Microglia-Mediated Neurodegeneration. J Biol Chem (2017) 292:13415–27. doi: 10.1074/jbc.M117.784983 PMC5555200 28655763

[50] Truong R Thankam FG Agrawal DK. Immunological Mechanisms Underlying Sterile Inflammation in the Pathogenesis of Atherosclerosis: Potential Sites for Intervention. Expert Rev Clin Immunol (2021) 17:37–50. doi: 10.1080/1744666x.2020.1860757 33280442 PMC7906938

[51] Liu D Yan J Sun J Liu B Ma W Li Y. BCL6 Controls Contact-Dependent Help Delivery During Follicular T-B Cell Interactions. Immunity (2021) 54:2245–55.e4. doi: 10.1016/j.immuni.2021.08.003 PMC8528402 34464595

[52] Fontenot JD Gavin MA Rudensky AY. Foxp3 Programs the Development and Function of CD4+CD25+ Regulatory T Cells. Nat Immunol (2003) 4:330–6. doi: 10.1038/ni904 12612578

[53] Chen J López-Moyado IF Seo H Lio CJ Hempleman LJ Sekiya T. NR4A Transcription Factors Limit CAR T Cell Function in Solid Tumours. Nature (2019) 567:530–4. doi: 10.1038/s41586-019-0985-x PMC6546093 30814732

[54] Zhou T Cheng J Yang P Wang Z Liu C Su X. Inhibition of Nur77/Nurr1 Leads to Inefficient Clonal Deletion of Self-Reactive T Cells. J Exp Med (1996) 183:1879–92. doi: 10.1084/jem.183.4.1879 PMC2192482 8666944

[55] Vlachos IS Paraskevopoulou MD Karagkouni D Georgakilas G Vergoulis T Kanellos I. DIANA-TarBase V7.0: Indexing More Than Half a Million Experimentally Supported miRNA:mRNA Interactions. Nucleic Acids Res (2015) 43:D153–9. doi: 10.1093/nar/gku1215 PMC4383989 25416803

[56] Chou CH Chang NW Shrestha S Hsu SD Lin YL Lee WH. Mirtarbase 2016: Updates to the Experimentally Validated miRNA-Target Interactions Database. Nucleic Acids Res (2016) 44:D239–47. doi: 10.1093/nar/gkv1258 PMC4702890 26590260

[57] Migita K Miyashita T Maeda Y Nakamura M Yatsuhashi H Kimura H. Toll-Like Receptor Expression in Lupus Peripheral Blood Mononuclear Cells. J Rheumatol (2007) 34:493–500. 17295441

[58] van der Houwen TB Dik WA Goeijenbier M Hayat M Nagtzaam NMA van Hagen M. Leukocyte Toll-Like Receptor Expression in Pathergy Positive and Negative Behçet’s Disease Patients. Rheumatol (Oxf Engl) (2020) 59:3971–9. doi: 10.1093/rheumatology/keaa251 PMC7733715 32756992

[59] Agalave NM Larsson M Abdelmoaty S Su J Baharpoor A Lundback P. Spinal HMGB1 Induces TLR4-Mediated Long-Lasting Hypersensitivity and Glial Activation and Regulates Pain-Like Behavior in Experimental Arthritis. Pain (2014) 155:1802–13. doi: 10.1016/j.pain.2014.06.007 24954167

[60] Fillatreau S Manfroi B Dörner T. Toll-Like Receptor Signalling in B Cells During Systemic Lupus Erythematosus. Nat Rev Rheumatol (2021) 17:98–108. doi: 10.1038/s41584-020-00544-4 33339987 PMC7747191

[61] Vollmer J Tluk S Schmitz C Hamm S Jurk M Forsbach A. Immune Stimulation Mediated by Autoantigen Binding Sites Within Small Nuclear RNAs Involves Toll-Like Receptors 7 and 8. J Exp Med (2005) 202:1575–85. doi: 10.1084/jem.20051696 PMC2213330 16330816

[62] Gong T Liu L Jiang W Zhou R. DAMP-Sensing Receptors in Sterile Inflammation and Inflammatory Diseases. Nat Rev Immunol (2020) 20:95–112. doi: 10.1038/s41577-019-0215-7 31558839

[63] Soni C Perez OA Voss WN Pucella JN Serpas L Mehl J. Plasmacytoid Dendritic Cells and Type I Interferon Promote Extrafollicular B Cell Responses to Extracellular Self-DNA. Immunity (2020) 52:1022–1038.e7. doi: 10.1016/j.immuni.2020.04.015 32454024 PMC7306002

[64] Ye J Wang YD Liu X Li LY Opejin A Hsueh EC. TLR7 Signaling Regulates Th17 Cells and Autoimmunity: Novel Potential for Autoimmune Therapy. J Immunol (2017) 199:941–54. doi: 10.4049/jimmunol.1601890 PMC5542577 28652396

[65] Tao K Fujii M Tsukumo S Maekawa Y Kishihara K Kimoto Y. Genetic Variations of Toll-Like Receptor 9 Predispose to Systemic Lupus Erythematosus in Japanese Population. Ann Rheum Dis (2007) 66:905–9. doi: 10.1136/ard.2006.065961 PMC1955115 17344245

[66] Celhar T Yasuga H Lee HY Zharkova O Tripathi S Thornhill SI. Toll-Like Receptor 9 Deficiency Breaks Tolerance to RNA-Associated Antigens and Up-Regulates Toll-Like Receptor 7 Protein in Sle1 Mice. Arthritis Rheumatol (2018) 70:1597–609. doi: 10.1002/art.40535 PMC6175219 29687651

[67] Tran NL Manzin-Lorenzi C Santiago-Raber ML. Toll-Like Receptor 8 Deletion Accelerates Autoimmunity in a Mouse Model of Lupus Through a Toll-Like Receptor 7-Dependent Mechanism. Immunology (2015) 145:60–70. doi: 10.1111/imm.12426 25424423 PMC4405324

[68] Santiago-Raber ML Dunand-Sauthier I Wu T Li QZ Uematsu S Akira S. Critical Role of TLR7 in the Acceleration of Systemic Lupus Erythematosus in TLR9-Deficient Mice. J Autoimmun (2010) 34:339–48. doi: 10.1016/j.jaut.2009.11.001 19944565

[69] Christensen SR Shupe J Nickerson K Kashgarian M Flavell RA Shlomchik MJ. Toll-Like Receptor 7 and TLR9 Dictate Autoantibody Specificity and Have Opposing Inflammatory and Regulatory Roles in a Murine Model of Lupus. Immunity (2006) 25:417–28. doi: 10.1016/j.immuni.2006.07.013 16973389

[70] Wu X Peng SL. Toll-Like Receptor 9 Signaling Protects Against Murine Lupus. Arthritis Rheum (2006) 54:336–42. doi: 10.1002/art.21553 16385525

[71] Vobořil M Brabec T Dobeš J Šplíchalová I Březina J Čepková A. Toll-Like Receptor Signaling in Thymic Epithelium Controls Monocyte-Derived Dendritic Cell Recruitment and Treg Generation. Nat Commun (2020) 11:2361. doi: 10.1038/s41467-020-16081-3 32398640 PMC7217920

[72] Nickerson KM Christensen SR Shupe J Kashgarian M Kim D Elkon K. TLR9 Regulates TLR7- and MyD88-Dependent Autoantibody Production and Disease in a Murine Model of Lupus. J Immunol (Baltimore Md 1950) (2010) 184:1840–8. doi: 10.4049/jimmunol.0902592 PMC4098568 20089701

[73] Suthers AN Sarantopoulos S. TLR7/TLR9- and B Cell Receptor-Signaling Crosstalk: Promotion of Potentially Dangerous B Cells. Front Immunol (2017) 8:775. doi: 10.3389/fimmu.2017.00775 28751890 PMC5507964

[74] Tabeta K Hoebe K Janssen EM Du X Georgel P Crozat K. The Unc93b1 Mutation 3d Disrupts Exogenous Antigen Presentation and Signaling *via* Toll-Like Receptors 3, 7 and 9. Nat Immunol (2006) 7:156–64. doi: 10.1038/ni1297 16415873

[75] Majer O Liu B Woo BJ Kreuk LSM Van Dis E Barton GM. Release From UNC93B1 Reinforces the Compartmentalized Activation of Select TLRs. Nature (2019) 575:371–4. doi: 10.1038/s41586-019-1611-7 PMC6856438 31546247

[76] Majer O Liu B Kreuk LSM Krogan N Barton GM. UNC93B1 Recruits Syntenin-1 to Dampen TLR7 Signalling and Prevent Autoimmunity. Nature (2019) 575:366–70. doi: 10.1038/s41586-019-1612-6 PMC6856441 31546246

[77] Sasai M Iwasaki A. Love Triangle Between Unc93B1, TLR7, and TLR9 Prevents Fatal Attraction. Immunity (2011) 35:3–5. doi: 10.1016/j.immuni.2011.07.006 21777792 PMC3143494

[78] Fukui R Saitoh S Matsumoto F Kozuka-Hata H Oyama M Tabeta K. Unc93B1 Biases Toll-Like Receptor Responses to Nucleic Acid in Dendritic Cells Toward DNA- But Against RNA-Sensing. J Exp Med (2009) 206:1339–50. doi: 10.1084/jem.20082316 PMC2715051 19451267

[79] Arleevskaya MI Larionova RV Brooks WH Bettacchioli E Renaudineau Y. Toll-Like Receptors, Infections, and Rheumatoid Arthritis. Clin Rev Allergy Immunol (2020) 58:172–81. doi: 10.1007/s12016-019-08742-z 31144208

[80] Saadoun D Garrido M Comarmond C Desbois AC Domont F Savey L. Th1 and Th17 Cytokines Drive Inflammation in Takayasu Arteritis. Arthritis Rheumatol (Hoboken NJ) (2015) 67:1353–60. doi: 10.1002/art.39037 25604824

[81] Pan LL Du J Gao N Liao H Wan J Ci WP. IL-9-Producing Th9 Cells may Participate in Pathogenesis of Takayasu’s Arteritis. Clin Rheumatol (2016) 35:3031–6. doi: 10.1007/s10067-016-3399-2 27629397

[82] Desbois AC Régnier P Quiniou V Lejoncour A Maciejewski-Duval A Comarmond C. Specific Follicular Helper T Cell Signature in Takayasu Arteritis. Arthritis Rheumatol (Hoboken NJ) (2021) 73:1233–43. doi: 10.1002/art.41672 33538119

[83] Gao N Cui W Zhao LM Li TT Zhang JH Pan LL. Contribution of Th2-Like Treg Cells to the Pathogenesis of Takayasu’s Arteritis. Clin Exp Rheumatol (2020) 38 Suppl:124 48–54. 31969221

[84] Barish GD Yu RT Karunasiri M Ocampo CB Dixon J Benner C. Bcl-6 and NF-kappaB Cistromes Mediate Opposing Regulation of the Innate Immune Response. Genes Dev (2010) 24:2760–5. doi: 10.1101/gad.1998010 PMC3003193 21106671

[85] Park HY Go H Song HR Kim S Ha GH Jeon YK. Pellino 1 Promotes Lymphomagenesis by Deregulating BCL6 Polyubiquitination. J Clin Invest (2014) 124:4976–88. doi: 10.1172/jci75667 PMC4347227 25295537

[86] Figueroa-Hall LK Paulus MP Savitz J. Toll-Like Receptor Signaling in Depression. Psychoneuroendocrinology (2020) 121:104843. doi: 10.1016/j.psyneuen.2020.104843 32911436 PMC7883590

[87] He X Jing Z Cheng G. MicroRNAs: New Regulators of Toll-Like Receptor Signalling Pathways. BioMed Res Int (2014) 2014:945169. doi: 10.1155/2014/945169 24772440 PMC3977468

[88] Piriyapongsa J Jordan IK. A Family of Human microRNA Genes From Miniature Inverted-Repeat Transposable Elements. PloS One (2007) 2:e203. doi: 10.1371/journal.pone.0000203 17301878 PMC1784062

[89] Liang T Guo L Liu C. Genome-Wide Analysis of Mir-548 Gene Family Reveals Evolutionary and Functional Implications. J Biomed Biotechnol (2012) 2012:679563. doi: 10.1155/2012/679563 23091353 PMC3468316

[90] Xu Y Zhong YD Zhao XX. MiR-548b Suppresses Proliferative Capacity of Colorectal Cancer by Binding WNT2. Eur Rev Med Pharmacol Sci (2020) 24:10535–41. doi: 10.26355/eurrev_202010_23406 33155209

[91] Sha MX Huang XW Yin Q. MiR-548b-3p Inhibits Proliferation and Migration of Breast Cancer Cells by Targeting MDM2. Eur Rev Med Pharmacol Sci (2020) 24:3105–12. doi: 10.26355/eurrev_202003_20675 32271428

[92] Pan Y Liang W Zhao X Liu L Qing Y Li Y. miR-548b Inhibits the Proliferation and Invasion of Malignant Gliomas by Targeting Metastasis Tumor-Associated Protein-2. Neuroreport (2016) 27:1266–73. doi: 10.1097/wnr.0000000000000690 27682888

[93] Qiu H Zhang G Song B Jia J. MicroRNA-548b Inhibits Proliferation and Invasion of Hepatocellular Carcinoma Cells by Directly Targeting Specificity Protein 1. Exp Ther Med (2019) 18:2332–40. doi: 10.3892/etm.2019.7812 PMC6704539 31452716

[94] Lin L Wang Y. miR-548b-3p Regulates Proliferation, Apoptosis, and Mitochondrial Function by Targeting CIP2A in Hepatocellular Carcinoma. BioMed Res Int (2018) 2018:7385426. doi: 10.1155/2018/7385426 30671469 PMC6323450

[95] Feng XE. miR-548b Suppresses Melanoma Cell Growth, Migration, and Invasion by Negatively Regulating Its Target Gene Hmgb1. Cancer Biother Radiopharm (2021) 36:189–201. doi: 10.1089/cbr.2019.3507 33750228

[96] Jin M Lu S Wu Y Yang C Shi C Wang Y. Hsa_circ_0001944 Promotes the Growth and Metastasis in Bladder Cancer Cells by Acting as a Competitive Endogenous RNA for miR-548. J Exp Clin Cancer Res CR (2020) 39:186. doi: 10.1186/s13046-020-01697-6 32928266 PMC7490907

[97] Takashima Y Kawaguchi A Iwadate Y Hondoh H Fukai J Kajiwara K. miR-101, miR-548b, miR-554, and miR-1202 Are Reliable Prognosis Predictors of the miRNAs Associated With Cancer Immunity in Primary Central Nervous System Lymphoma. PloS One (2020) 15:e0229577. doi: 10.1371/journal.pone.0229577 32101576 PMC7043771

[98] Oshiumi H. Circulating Extracellular Vesicles Carry Immune Regulatory miRNAs and Regulate Vaccine Efficacy and Local Inflammatory Response After Vaccination. Front Immunol (2021) 12:685344. doi: 10.3389/fimmu.2021.685344 34211472 PMC8239358

[99] Tsukamoto H Kouwaki T Oshiumi H. Aging-Associated Extracellular Vesicles Contain Immune Regulatory microRNAs Alleviating Hyperinflammatory State and Immune Dysfunction in the Elderly. iScience (2020) 23:101520. doi: 10.1016/j.isci.2020.101520 32927264 PMC7495115

[100] Rosenberger CM Podyminogin RL Navarro G Zhao GW Askovich PS Weiss MJ. miR-451 Regulates Dendritic Cell Cytokine Responses to Influenza Infection. J Immunol (Baltimore Md 1950) (2012) 189:5965–75. doi: 10.4049/jimmunol.1201437 PMC3528339 23169590

[101] Miyashita Y Ishikawa K Fukushima Y Kouwaki T Nakamura K Oshiumi H. Immune-Regulatory microRNA Expression Levels Within Circulating Extracellular Vesicles Correspond With the Appearance of Local Symptoms After Seasonal Flu Vaccination. PloS One (2019) 14:e0219510. doi: 10.1371/journal.pone.0219510 31287847 PMC6615615

[102] Jin MS Kim SE Heo JY Lee ME Kim HM Paik S-G. Crystal Structure of the TLR1-TLR2 Heterodimer Induced by Binding of a Tri-Acylated Lipopeptide. Cell (2007) 130:1071–82. doi: 10.1016/j.cell.2007.09.008 17889651

[103] Mukherjee A Roy S Patidar A Bodhale N Dandapat J Saha B. TLR2 Dimer-Specific Ligands Selectively Activate Protein Kinase C Isoforms in Leishmania Infection. Immunology (2021) 164:318–31. doi: 10.1111/imm.13373 PMC8442242 34021910

[104] Spitzer JH Visintin A Mazzoni A Kennedy MN Segal DM. Toll-Like Receptor 1 Inhibits Toll-Like Receptor 4 Signaling in Endothelial Cells. Eur J Immunol (2002) 32:1182–7. doi: 10.1002/1521-4141(200204)32:4<1182::Aid-immu1182>3.0.Co;2-9 11932926

[105] da Rocha Sobrinho HM Saar Gomes R da Silva DJ Quixabeira VBL Joosten LAB Ribeiro de Barros Cardoso C. Toll-Like Receptor 10 Controls TLR2-Induced Cytokine Production in Monocytes From Patients With Parkinson’s Disease. J Neurosci Res (2021) 99:2511–24. doi: 10.1002/jnr.24916 34260774

[106] Sun J Wiklund F Zheng SL Chang B Bälter K Li L. Sequence Variants in Toll-Like Receptor Gene Cluster (TLR6-TLR1-TLR10) and Prostate Cancer Risk. J Natl Cancer Inst (2005) 97:525–32. doi: 10.1093/jnci/dji070 15812078

