# Supplementary material for: Identification of the Association Between Toll-Like Receptors and T-Cell Activation in Takayasu’s Arteritis
Source: Front Immunol. 2022 Jan 20;12:792901. doi: 10.3389/fimmu.2021.792901 (PMC8812403; doi:10.3389/fimmu.2021.792901)
Supplement: Supplementary file 1 [file Table_1.pdf]

Supplementary Table 1 Primers used in real-time fluorescence quantitative polymerase chain reaction

Gene symbol	NCBI Reference Sequence	Forward primer	Reverse primer
CD83	NC_000006.12	GAGAAACCTAAGTGGCAAGGTG	AGGACAATCTCCGCTCTGTAT
CCL5	NC_000017.11	CCAGCAGTCGTCTTTGTCAC	CTCTGGGTTGGCACACACTT
TLR1	NC_000004.12	TTCAAACGTGAAGCTACAGGG	CCGAACACATCGCTGACAACT
TLR2	NC_000004.12	ATCCTCCAATCAGGCTTCTCT	GGACAGGTCAAGGCTTTTTACA
TLR4	NC_000009.12	AGACCTGTCCCTGAACCCTAT	CGATGGACTTCTAAACCAGCCA
TLR6	NC_000004.12	TTCTCCGACGGAAATGAATTTGC	CAGCGGTAGGTCTTTTGGAAC
TLR8	NC_000023.11	ATGTTCCTTCAGTCGTCAATGC	TTGCTGCACTCTGCAATAACT
P50	NC_000004.12	AACAGAGAGGATTTCGTTTCCG	TTTGACCTGAGGGTAAGACTTCT
P65	NC_000011.10	GAAGAAGAGTCCTTTCAGCG	GGGATGACGTAAAGGGATAG
IκBα	NC_000014.9	TCCACTCCATCCTGAAGGCTACCAA	GACATCAGCACCCAAGGACACCAAA
TNF-α	NC_000006.12	GGAGAAGGGTGACCGACTCA	CTGCCCAGACTCGGCAA
PDCD1	NC_000002.12	GTGCCTGTGTTCTCTGTGGA	TCCGCTAGGAAAGACAATGG
PD-L1	NC_000009.12	GGTTGTGGATCCAGTCACCT	TTGGTGGTGGTGGTCTTACC
PD-L2	NC_000009.12	ACCGTGAAAGAGCCACTTTG	GCGACCCCATAGATGATTATGC

CTLA4	NC_000002.12	CATGATGGGGAATGAGTTGACC	TCAGTCCTTGGATAGTGAGGTTC
TIM3	NC_000005.10	CTGCTGCTACTACTTACAAGGTC	GCAGGGCAGATAGGCATTCT
LAG3	NC_000012.12	GCGGGGACTTCTCGCTATG	GGCTCTGAGAGATCCTGGGG
TIGIT	NC_000003.12	ATACAAGAGCGAAGGTCTCACG	CTGAGTCTCCCATAACAGCGG
CD28	NC_000002.12	CTATTTCCCGGACCTTCTAAGCC	GCGGGGAGTCATGTTCATGTA
CD40	NC_000020.11	TTGGGGTCAAGCAGATTGCTA	GCAGATGACACATTGGAGAAGA
CD40L	NC_000023.11	ACATACAACCAAACTTCTCCCCG	GCAAAAAGTGCTGACCCAATCA
TCR	NC_000014.9	CCTTCAACAACAGCATTATTATTCCAG	CGAGGGAGCACAGGCTGTCTTA
CD3	NC_000001.11	GCCAGAACCAGCTCTATAAC	GGCCACGTCTCTTGTCCAA
T-bet	NC_000017.11	TGACCCAGATGATTGTGCTCCAGT	AATCTCGGCATTCTGGTAGGCAGT
GATA3	NC_000010.11	CACCACAACCACACTCTG	GCCTTCCTTCTTCATAGTCA
RORC	NC_000001.11	GTGGGGACAAGTCGTCTGG	AGTGCTGGCATCGGTTTCG
BCL6	NC_000003.12	GGAGTCGAGACATCTTGACTGA	ATGAGGACCGTTTTATGGGCT
FOXP3	NC_000023.11	GTGGCCCGGATGTGAGAAG	GGAGCCCTTGTCGGATGATG
GAPDH	NC_000012.12	GCACCGTCAAGGCTGAGAAC	ATGGTGGTGAAGACGCCAGT
ACTB	NC_000007.14	CCACCATGTACCCTGGCATT	ACTCCTGCTTGCTGATCCAC

β-glucuronidase	NC_000007.14	GACACGCTAGAGCATGAGGG	GGGTGAGTGTGTTGTTGATGG
SDHA	NC_000005.10	CAGCATGTGTTACCAAGCTGT	GGTGTCGTAGAAATGCCACCT
HPRT1	NC_000023.11	TTTATTCCTCATGGACTAATTATGGA	CCTCCCATCTCCTTCATCAC
RPL13A	NC_000019	AAAAGCGGATGGTGGTTCCT	GCTGTCACTGCCTGGTACTT
B2M	NC_000015.10	GAGGCTATCCAGCGTACTCCA	CGGCAGGCATACTCATCTTTT
YWHAZ	NC_000008.11	AGACGGAAGGTGCTGAGAAA	CGTTGGGGATCAAGAACTTT
PKG1	NC_000023.11	TGGACGTTAAAGGGAAGCGG	GCTCATAAGGACTACCGACTTGG

Abbreviation: *CCL5*, C-C motif chemokine ligand 5. *TLR1*, toll like receptor 1. *p50*, nuclear factor kappa B (NFκB) subunit 1. *p65*, RELA proto-oncogene NFκB subunit. IκBα, NFκB inhibitor alpha. *TNF*, tumor necrosis factor. *PDCD1*, programmed cell death 1, also known as *PD-1*. *PD-L1*, *CD274*. *PD-L2*, programmed cell death 1 ligand 2. *CTLA4*, cytotoxic T-lymphocyte associated protein 4. *TIM3*, hepatitis A virus cellular receptor 2. *LAG3*, lymphocyte activating 3. *TIGIT*, TNF superfamily member 14. *TCR*, T cell receptor. *T-bet*, T-box transcription factor 21. *GATA3*, GATA binding protein 3. *RORC*, RAR related orphan receptor C. *BCL6*, BCL6 transcription repressor. *FOXP3*, forkhead box P3. *GAPDH*, glyceraldehyde-3-phosphate dehydrogenase. *ACTB*, β-actin. *β-glucuronidase*. *SDHA*, succinate dehydrogenase complex flavoprotein subunit A A. *HPRT1*, hypoxanthine phosphoribosyl transferase 1. *RPL13A*, Ribosomal Protein L13a. *B2M*, beta-2-microglobulin. *YWHAZ*, yrosine 3-monooxygenase/tryptophan 5. *PKG1*, phosphoglycerate kinase 1.
