# Supplementary material for: Identification of the Association Between Toll-Like Receptors and T-Cell Activation in Takayasu’s Arteritis
Source: Front Immunol. 2022 Jan 20;12:792901. doi: 10.3389/fimmu.2021.792901 (PMC8812403; doi:10.3389/fimmu.2021.792901)
Supplement: Supplementary file 2 [file Table_2.pdf]

Supplementary Table 2 Demographic data and clinical features of patients with Takayasu's arteritis.

		Untreated (n=7)	Treated (n=20)	p-value[a]	Active-treated (n=11)	Inactive-treated (n=9)	p-value[b]
Age (year)		29.57±5.22	39.40±9.27	0.0093	39.36±7.89	39.44±11.24	0.82
Sex (male/ female)		2/5	1/19	0.088	1/10	0/9	0.35
Disease duration (months)		45.14±65.34	43.50(12.25,128.50)	0.15	118(13,200)	35.56±26.66	0.95
ESR (mm/h)		47.71±48.55	14.60±9.17	0.22	16.82±11.34	11.89±4.88	0.33
hs-CRP (mg/L)		11.28(0.32, 113.62)	1.00(0.44,5.85)	0.34	7.13±7.31	0.84(0.26,1.93)	0.02
Interleukin 6 (pg/mL)		6.30(2.00,22.20)	2.10(2.00,4.40)	0.16	4.27±2.65	2.00(2.00,2.70)	0.95
TNF-α (pg/mL)		4.59±2.56	6.10(5.60,8.00)	0.12	6.85(5.50,8.38)	6.60±2.24	0.66
Corticosteroid	used/ non-used	0/7	18/2	0.000014	10/1	8/1	0.88
	Dose(mg/d)[a]	0	10.00(8.125,32.25)	0.00014	10(10,40)	10.00(6.25,29.50)	0.37

a Untrated Vs. Treated. b Inactive-treated Vs. Active-treated. Ref. range, reference range. M, male. F, female. ESR, erythrocyte sedimentation rate. hs-CRP, hypersensitive- C reactive protein. TNF-α., Tumor necrosis factor-α.
