# Supplementary material for: Identification of the Association Between Toll-Like Receptors and T-Cell Activation in Takayasu’s Arteritis
Source: Front Immunol. 2022 Jan 20;12:792901. doi: 10.3389/fimmu.2021.792901 (PMC8812403; doi:10.3389/fimmu.2021.792901)
Supplement: Supplementary file 5 [file Table_5.pdf]

Supplementary Table 5 Assessment for the disease activity of the TAK using TLRs and their correlation cluster.

Patient	TLR8-CCL5	TLR8-Tbet	TLR4-CCL5	TLR6-CCL5	Total score
# 1	0	0	0	1	1
# 2	0	0	0	0	0
# 3	0	0	0	0	0
# 4	0	0	0	0	0
# 5	0	0	0	0	0
# 6	0	0	0	0	0
# 7	0	0	0	0	0
# 8	0	0	0	0	0
# 9	0	0	0	0	0
# 10	0	0	0	1	1
# 11	1	1	1	0	3
# 12	1	0	1	1	3
# 13	1	1	1	1	4
# 14	1	1	1	1	4
# 15	0	0	0	1	1
# 16	0	0	0	0	0
# 17	1	0	1	1	3
# 18	1	1	1	1	4
# 19	1	1	1	1	4
# 20	0	1	1	1	3
**Threshold**	0.349929867	0.63839009	0.348022575	0.148140708	0.5
**Sensitivity**	63.60%	54.50%	72.70%	90%	88.90%
**Specificity**	100%	100%	100%	80%	90.90%
**Positive predictive value**	100%	100%	100%	81.80%	88.90%
**Negative predictive value**	60%	64.30%	75%	88.90%	90.90%
**Regression equation**	y=-6.54855x+10.45201	y=-1.21735x+1.470437	y=-5.42142x+9.938277	y=-23.4056x+10.78878	-
r	-0.818	-0.837	-0.878	-0.903	-
**Pearson's *p*-value**	0.0070	0.0049	0.0018	0.0009	-
**AUC**	0.828	0.778	0.848	0.818	0.939
