# Supplementary material for: Identification of the Association Between Toll-Like Receptors and T-Cell Activation in Takayasu’s Arteritis
Source: Front Immunol. 2022 Jan 20;12:792901. doi: 10.3389/fimmu.2021.792901 (PMC8812403; doi:10.3389/fimmu.2021.792901)
Supplement: Supplementary file 6 [file Table_6.pdf]

Supplementary Table 6 Assessment for the disease activity of the TAK using TLR4-TLR6 gene pair.

Patient	TLR6-TLR4
# 1	0
# 2	0
# 3	0
# 4	0
# 5	0
# 6	0
# 7	0
# 8	0
# 9	0
# 10	1
# 11	1
# 12	0
# 13	1
# 14	1
# 15	1
# 16	1
# 17	1
# 18	1
# 19	1
# 20	1
**Threshold**	0.173343259
**Sensitivity**	90.90%
**Specificity**	100%
**Positive predictive value**	100%
**Negative predictive value**	90%
**Regression equation**	y=4.000846x-0.09562
**R**	-0.953
**Pearson's P-value**	0.0001
**AUC**	0.919
