# Supplementary material for: Identification of the Association Between Toll-Like Receptors and T-Cell Activation in Takayasu’s Arteritis
Source: Front Immunol. 2022 Jan 20;12:792901. doi: 10.3389/fimmu.2021.792901 (PMC8812403; doi:10.3389/fimmu.2021.792901)
Supplement: Supplementary file 7 [file Table_7.pdf]

Supplementary Table 7 The detailed evaluation the closeness of gene-to-gene functional relationships between TLRs and the other genes.

Pearson correlation					Spearman correlation				
Genes	Co-expressed TLR-genes	Common neighborhoods with TLRs	TLRs in the co-expression cluster	Score	Genes	Co-expressed TLR-genes	Common neighborhoods with TLRs	TLRs in the co-expression cluster	Score
TAK patients with inactive disease									
BCL6	TLR1, TLR2, TLR4, TLR6	FOXP3, CCL5, NR4A1, CD28	TLR1, TLR4, TLR6, TLR8	12	BCL6	TLR1, TLR2, TLR4, TLR6	FOXP3, IκBα, TIGIT, CCL5, NR4A1, CD28, GATA3, TCR	TLR1, TLR2, TLR4, TLR6	16
CCL5	TLR1, TLR2, TLR4, TLR6. TLR8	BCL6, CD28, CD3, FOXP3, GATA3, T-bet	-	11	TIGIT	TLR1, TLR2, TLR4, TLR6	BCL6, CD28, FOXP3, IκBα, TCR, NR4A1	TLR1, TLR2, TLR4, TLR6	14
FOXP3	TLR1, TLR4, TLR6	BCL6, CCL5, NR4A1, CD28	-	7	IκBα	TLR1, TLR2, TLR4, TLR6	BCL6, FOXP3, TIGIT, TNF, NR4A1	TLR1, TLR2, TLR4, TLR6	13
GATA3	TLR2, TLR6, TLR8	CCL5, CD28, T-bet, CD3	-	7	NR4A1	TLR1, TLR6	BCL6, CD83, FOXP3, IκBα, TIGIT, TNF	TLR1, TLR2, TLR4, TLR6	12
CD28	TLR4, TLR6	BCL6, FOXP3, CCL5, GATA3	-	6	FOXP3	TLR1, TLR4, TLR6	BCL6, CD28, CD83, IκBα, TCR, TIGIT, CCL5, NR4A1	-	11
T-bet	TLR4, TLR6, TLR8	CD3, GATA3, CCL5	-	6	CCL5	TLR1, TLR2, TLR4, TLR6, TLR8	BCL6, CD28, FOXP3, GATA3, T-bet	-	10
NR4A1	TLR1, TLR4, TLR6	BCL6, FOXP3	TLR2	6	CD28	TLR2, TLR4, TLR6	BCL6, FOXP3, TIGIT, CCL5, GATA3, TCR	-	9
CD3	TLR6	CCL5, GATA3, T-bet	-	4	GATA3	TLR2, TLR4, TLR6	BCL6, CD28, T-bet, CCL5	-	7
CD40	-	-	TLR1, TLR4, TLR6, TLR8	4	T-bet	TLR4, TLR6, TLR8	CCL5, GATA3	-	5
TIGIT	-	BCL6, CD28, NR4A1	TLR2	4	TCR	TLR2	BCL6, CD28, FOXP3, TIGIT	-	5

TCR	-	BCL6, CD28, FOXP3	-	3	CD40L	-	BCL6, CD28, FOXP3, TIGIT, NR4A1	-	5
CD40L	-	BCL6, FOXP3, CD28	-	3	CD83	TLR1	FOXP3, TNF, NR4A1	-	4
CTLA4	-	FOXP3, CD28	-	2	PD-L2	-	IκBα, TNF, NR4A1	TLR8	4
PD-1	TLR2	-	TLR2	2	LAG3	-	-	TLR1, TLR2, TLR4, TLR6	4
PD-L2	-	BCL6, FOXP3	-	2	TNF	TLR1	CD83, IκBα, NR4A1	-	4
TIM3	-	PD-1	TLR2	2	CD3	-	GATA3, T-bet	-	2
CD83	-	NR4A1	-	1	P50	-	IκBα, NR4A1	-	2
IκBα	-	NR4A1	-	1	CD40	-	CD83	-	1
P50	-	NR4A1	-	1	CTLA4	-	CD28	-	1
RORC	-	GATA3	-	1	PD-L1	-	CD83	-	1
LAG3	-	-	TLR2	1	P65	-	-	-	0
TNF	-	1	-	1	PD-1	-	-	-	0
P65	-	-	-	0	RORC	-	-	-	0
PD-L1	-	NR4A1	-	0	TIM3	-	-	-	0
TAK patients with active disease									
BCL6	TLR2, TLR4	-	TLR1, TLR2,TLR4, TLR6, TLR8	7	BCL6	-	LAG3	TLR1, TLR6	3
PD-1	-	BCL6	-	1	CD40	-	LAG3	TLR1, TLR6	3
LAG3	-	BCL6	-	1	LAG3	TLR6	-	TLR1, TLR6	3
CD28	-	-	-	0	PD-L2	-	-	TLR1, TLR6	2
CD3	-	-	-	0	CD83	TLR1	-	-	1
CD40	-	-	-	0	IκBα	TLR2	-	-	1
CD83	-	-	-	0	P50	-	IκBα	-	1
CTLA4	-	-	-	0	TIGIT	-	IκBα	-	1
FOXP3	-	-	-	0	TIM3	-	IκBα	-	1
GATA3	-	-	-	0	TNF	-	CD83	-	1
IκBα	-	-	-	0	NR4A1	-	CD83	-	1
P50	-	-	-	0	CD28	-	-	-	0

P65	-	-	-	0	CD3	-	-	-	0
PD-L1	-	-	-	0	CTLA4	-	-	-	0
PD-L2	-	-	-	0	FOXP3	-	-	-	0
RORC	-	-	-	0	GATA3	-	-	-	0
T-bet	-	-	-	0	P65	-	-	-	0
TCR	-	-	-	0	PD-1	-	-	-	0
TIGIT	-	-	-	0	PD-L1	-	-	-	0
TIM3	-	-	-	0	RORC	-	-	-	0
CCL5	-	-	-	0	T-bet	-	-	-	0
CD40L	-	-	-	0	TCR	-	-	-	0
TNF	-	-	-	0	CCL5	-	-	-	0
NR4A1	-	-	-	0	CD40L	-	-	-	0

Abbreviation: BCL6, BCL6 transcription repressor. CD3, CD247. PD-1, programmed cell death 1, also known as PDCD1.PD-L1, CD274. PD-L2, PDCD1LG2. LAG3, lymphocyte activating 3. CTLA4, cytotoxic T-lymphocyte associated protein 4. FOXP3, forkhead box P3. GATA3, GATA binding protein 3. IκBα, NFκB inhibitor alpha. NFKB1 NFKB1, nuclear factor kappa B (NFκB) subunit 1, also known as p50. RELA, RELA proto-oncogene NFκB subunit, also known as p65. PD-L1, CD274. PD-L2, programmed cell death 1 ligand 2 also known as PDCD1LG2. RORC, RAR related orphan receptor C. T-bet, T-box transcription factor 21, also known as TBX21. TCR, T cell receptor. TIGIT, TNF superfamily member 14. HAVCR2, hepatitis A virus cellular receptor 2, also known as TIM3. CCL5, C-C motif chemokine ligand 5. CD40L, CD40LG. TNF, tumor necrosis factor.TAK, Takayasu’s arteritis. TLRs, Toll-like receptors.
