# Supplementary material for: Identification of the Association Between Toll-Like Receptors and T-Cell Activation in Takayasu’s Arteritis
Source: Front Immunol. 2022 Jan 20;12:792901. doi: 10.3389/fimmu.2021.792901 (PMC8812403; doi:10.3389/fimmu.2021.792901)
Supplement: Supplementary file 10 [file DataSheet_1.pdf]

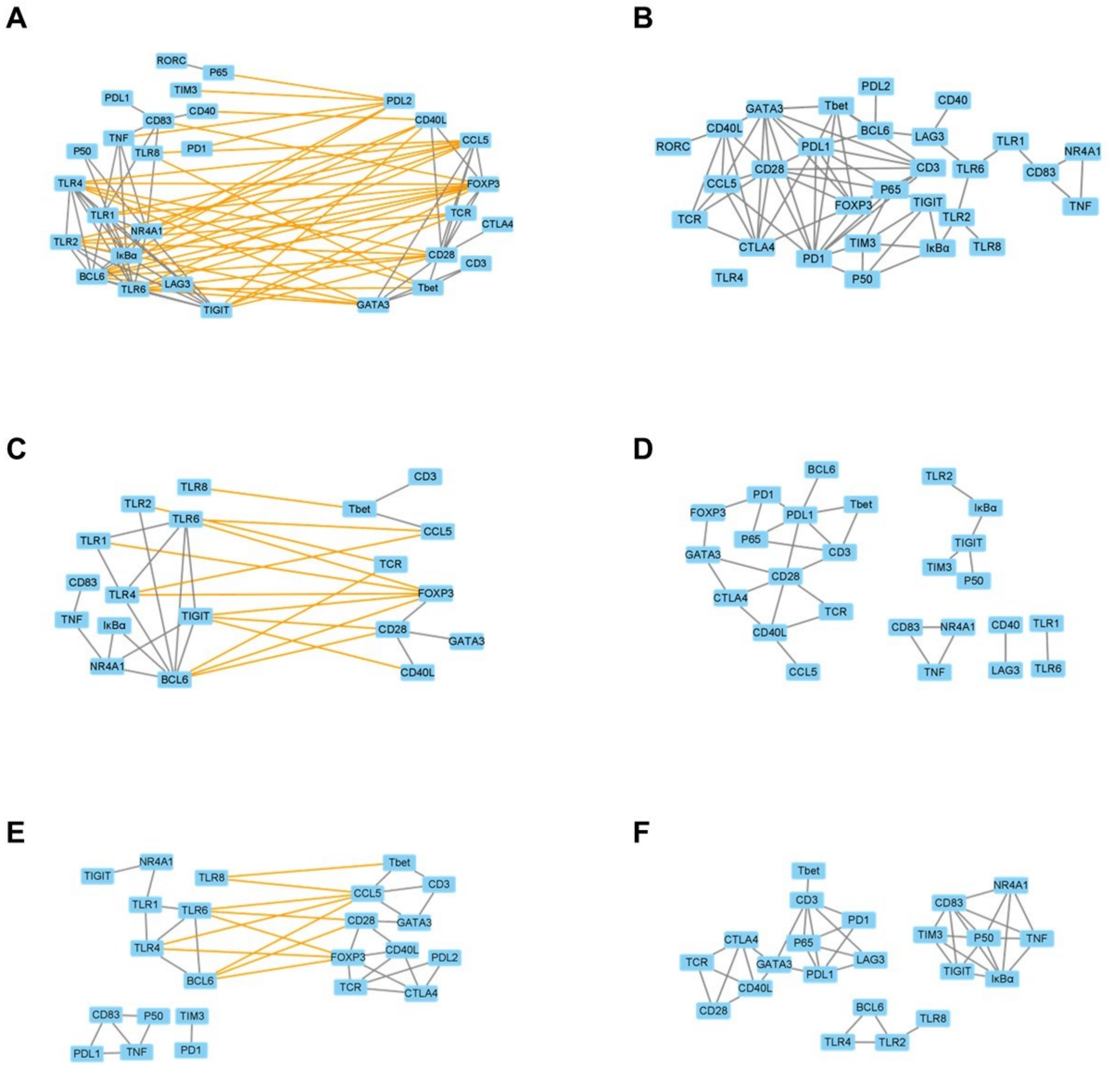

Supplementary Figure 1 The negative correlations between the TLRs gene cluster and the gene cluster associated with T-cell activation and differentiation in inactive-treated TAK. (A)~(B) The inactive-treated TAK group exhibited a unique pattern of inverse correlations between the TLRs gene cluster and the gene cluster associated with T-cell activation and differentiation, while the active-treated TAK group did not. **(C)~(F)** To more clearly demonstrate this feature, only the high correlations (defined as $|r| > 0.73$, $p < 0.01$) were showed. (A)~(D), Spearman correlation. (E)~(F), Pearson correlation. TAK, Takayasu's arteritis.

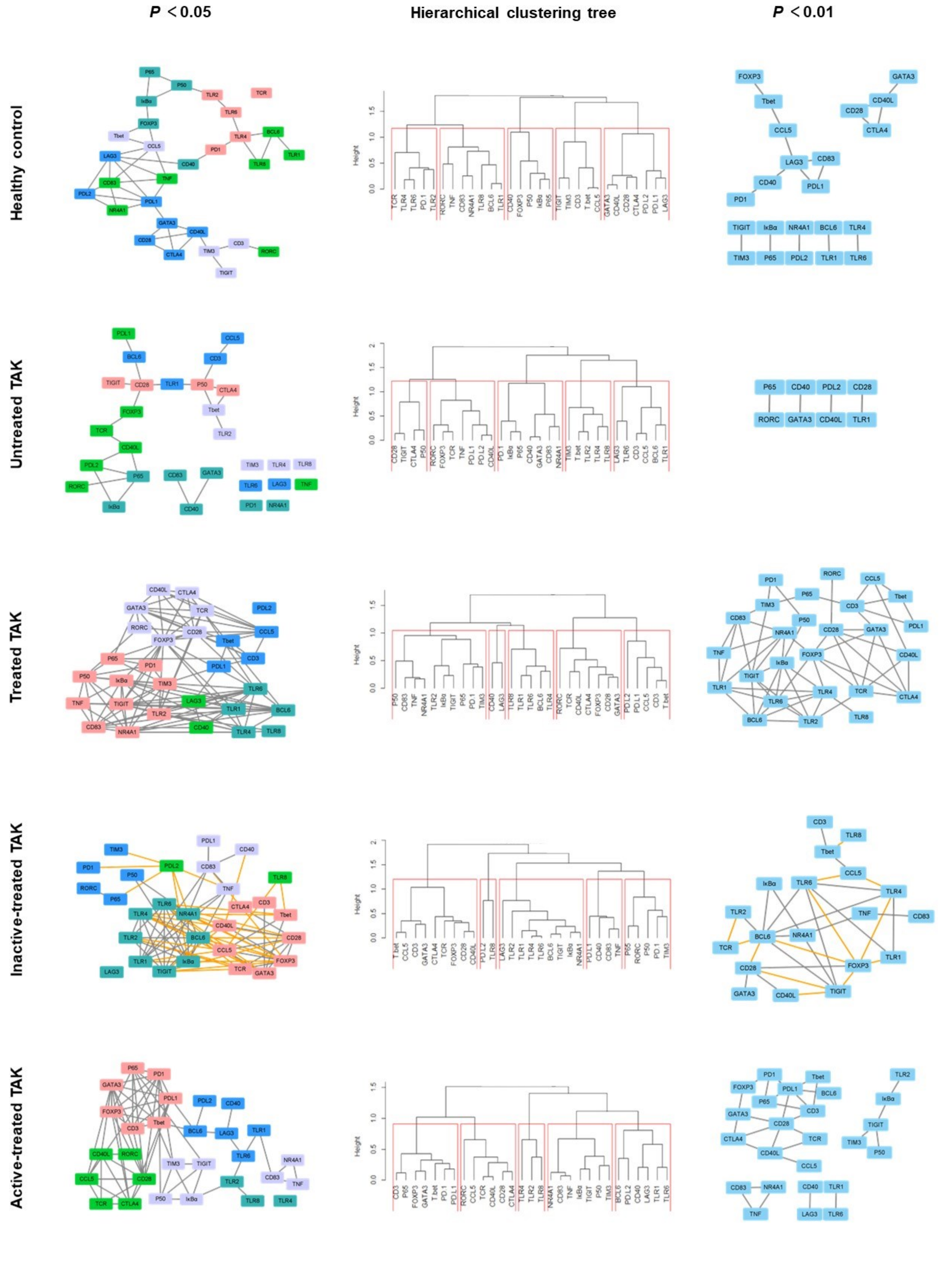

Supplementary Figure 2 Dynamic gene co-expression networks based on Spearman correlation.

Left panel, the gene co-expression networks consisting of correlation with a p-value less than 0.05. Right panel, to more clearly demonstrate this feature, only the high correlations (defined as $|r| > 0.73$, $p < 0.01$) were showed. Middle panel, the hierarchical clustering trees using complete method. As a result, genes were organized into 5 clusters by cutting the clustering tree at the height of 1.0, which was indicated by red frames. Genes belonging to the same cluster were like to have similar functions and were labeled the same color. The negative correlation was indicated by the yellow edge, while the positive correlation was grey in the networks.

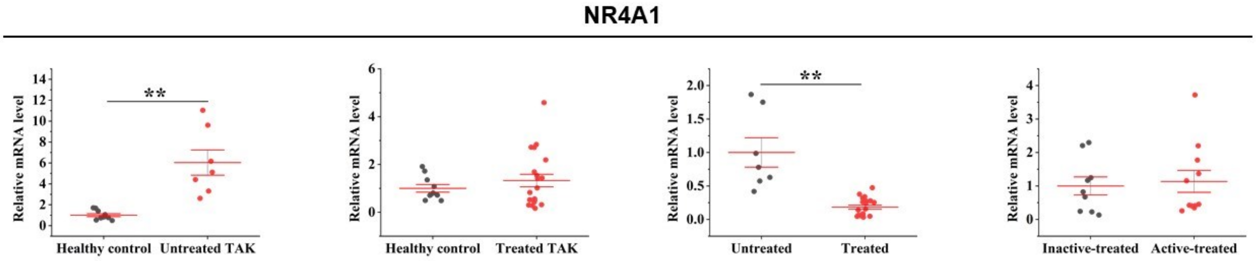

Supplementary Figure 3 Gene expression differential analysis of NR4A1. Mann–Whitney test. **$p < 0.01$. The red center line represented the mean value of the mRNA level, and the error bar showed the standard deviation. Healthy controls, n=10 people. Untreated TAK, n=7 people. Treated TAK, n=20 people. Active-treated TAK. n=11 people. Inactive-treated TAK, n=9 people. TAK, Takayasu's arteritis.

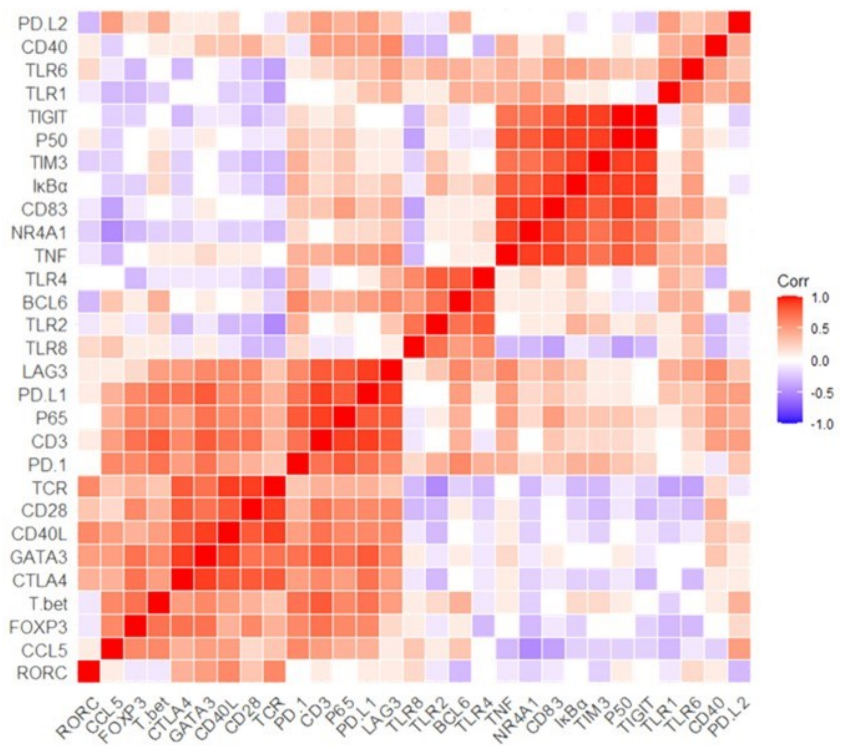

Supplementary Figure 4 Hierarchical clustering heatmap of the treated Takayasu's arteritis (TAK) group. The hierarchical clustering heatmap of the target genes with the distance calculated using Pearson correlation.

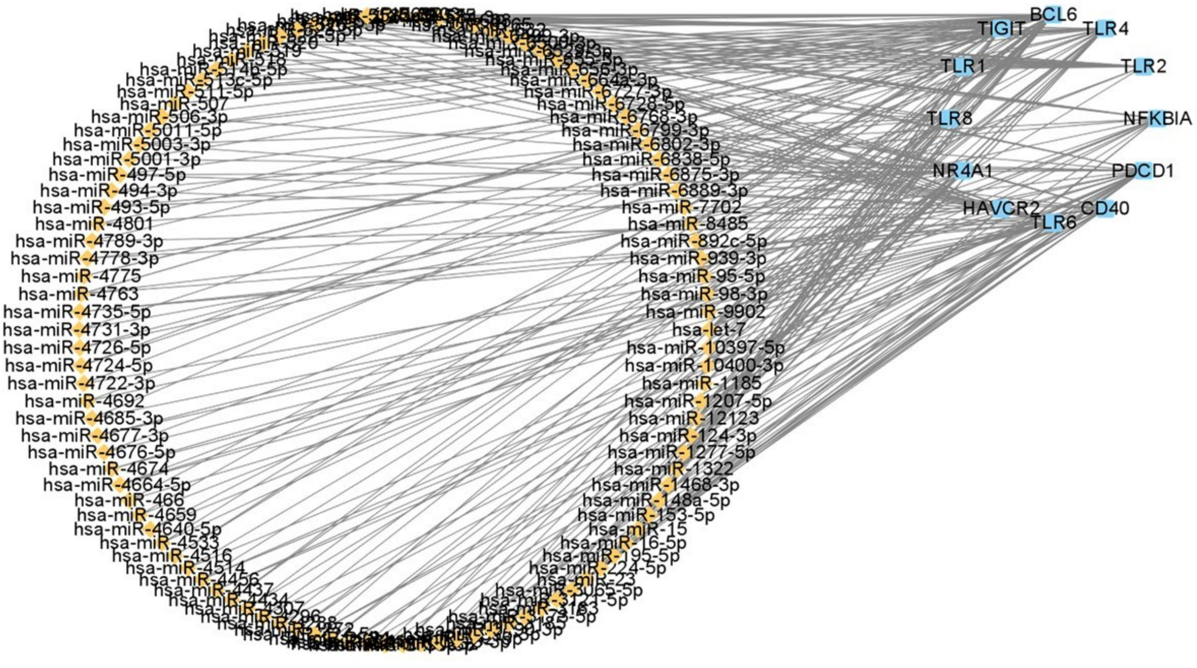

Supplementary Figure 5 The predicted miRNA-gene network related to the Toll-like receptors (TLRs) gene cluster in inactive-treated TAK. The predicted miRNA-gene network (based on miRDB database) which included the miRNAs with a degree greater than or equal to 2.
